# Obesity and the experienced cultural, economic and climate variations among immigrants to high-income countries: An umbrella review of the literature

**DOI:** 10.1017/S1368980026102687

**Published:** 2026-05-28

**Authors:** Mary Abed Al Ahad, Maya Kathryn Fenyk

**Affiliations:** Geography and Sustainable Development, University of St Andrews, UK

**Keywords:** Immigrant health, Obesity, Weight gain, Acculturation, Socio-economic determinants, High-income countries

## Abstract

**Objective:**

Obesity is a growing global public-health concern, particularly among immigrants to high-income countries. This umbrella literature review synthesises evidence from systematic reviews to explore the multifactorial cultural, socio-economic and environmental determinants of obesity and weight gain among immigrants to high-income countries.

**Design:**

A systematic search of PubMed, Web of Science and Scopus was conducted from January 2015 to November 2025 to identify systematic reviews and meta-analyses examining the impact of cultural, socio-economic and environmental factors on obesity among adult immigrants (i.e. economic, students and refugees) to high-income countries.

**Setting:**

Thirty-three reviews were included and thematically synthesised under four domains: physical climate and physiological changes, socio-economic stressors and psychological mediators, cultural factors and methodological quality – rated using the A Measurement Tool to Assess Systematic Reviews tool.

**Results:**

The review found consistent evidence linking longer duration of residence and higher levels of acculturation with increased obesity risk. Socio-economic disadvantage, food insecurity and psychological stress – particularly acculturation stress – were key mediators of weight gain. Less sunny colder climates impacted on weight gain indirectly through limiting physical activity and accessibility to traditional-healthy diet outlets. Cultural dietary transitions and intergenerational differences further influenced obesity trajectories. Methodological quality varied, with only six reviews providing meta-analysis pooled estimates of obesity and being rated as high to moderate quality.

**Conclusions:**

Obesity among immigrants is shaped by intersecting cultural, socio-economic, environmental and physiological factors. Public-health strategies must adopt culturally sensitive approaches that address structural barriers and support healthy transitions post-migration. Future research should emphasise longitudinal, intersectional and climate-tailored studies to inform targeted interventions.

## Graphical abstract

**Figure:**
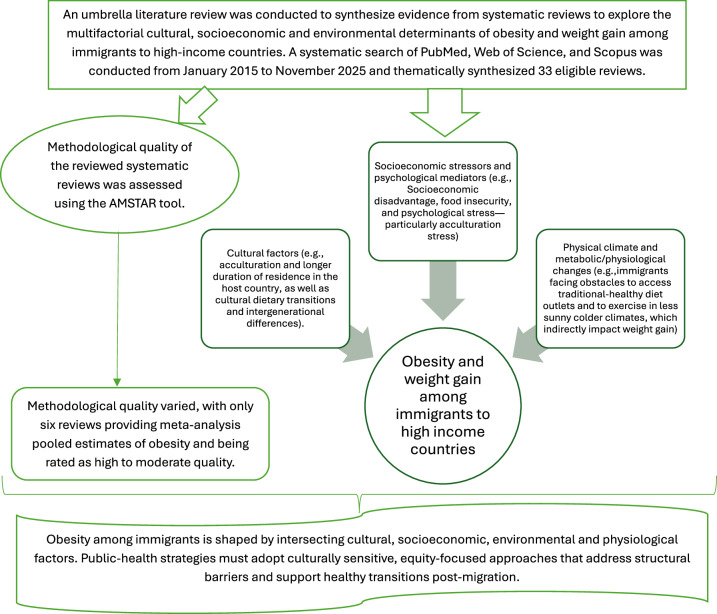

Obesity is a complex and growing public health issue characterised by an excessive accumulation of body fat, typically measured using the BMI. A BMI of 25 or higher is considered overweight and a BMI of 30 or higher is generally classified as obese^(1,2)^. The prevalence of obesity has been rising globally, affecting individuals of all ages, Sexes and socio-economic backgrounds, and it is considered to be the fifth highest risk factor for global deaths^(1,3)^. In 2021, higher-than-optimal BMI of more than 25 was linked to about 3·7 million deaths from noncommunicable diseases such as CVD, diabetes, cancers, musculoskeletal disorders, chronic respiratory diseases, digestive disorders, neurological disorders and mental health problems^(1,4)^. It is also estimated that over 60 % of adults in high-income countries fall into the overweight or obese categories^(5)^. For example, in England, around 64 % of adults are either overweight or obese^(5)^.

Whilst the etiology of obesity is multifactorial – encompassing biological, behavioural and environmental determinants, sociological/demographic circumstances such as migrating to a new country can also play a significant role in weight gain and obesity. As of 2020, approximately 3·6 % of the global population were classified as international migrants^(6)^. Immigrants to high-income countries (e.g. countries in North America and Europe) arrive initially in better health compared to the individuals born in the destination country due to upward positive selection (e.g. higher levels of education, skills, or economic resources), referred to as the Healthy Migrant Effect^(2,7–11)^. However, this health advantage declines over time as a result of shifting socio-economic, cultural and surrounding environmental factors - referred to as the Years Since Immigration Effect)^(2,7–12)^. Also, this health advantage declines due to the process of acculturation, specifically dietary acculturation whereby immigrants eventually acculturate to the – typically unhealthy – environments and dietary practices (e.g. high consumption of processed foods, fats, sugars and Na, and a concurrent decline in the intake of fruits, vegetables and fibre coupled with less physical activity and a sedentary behaviour) found in the destination country^(2,7–12)^. For example, a review by Elshahat and Moffat (2020) showed that acculturation of immigrants into a Western lifestyle include significant increase in consumption of low nutrient, energy-dense foods facilitated by personal barriers to healthful eating such as lack of nutrition awareness and language issues^(13)^. The concept of dietary acculturation is particularly noticeable with respect to CVD and related risk factors such as obesity, hypertension, diabetes and metabolic syndrome^(14)^. This would place immigrants at a disproportionately higher risk of developing obesity and related cardiometabolic conditions compared to domestic-born populations in the host countries^(14)^.

Additionally, immigrants might face psychological stresses as a result of adapting to a new cultural environment including anxiety, depression and social isolation, all of which have bidirectional relationships with nutritional status and obesity^(15)^. For example, a scoping review by Elshahat *et al.* (2023) found that diets rich in fruit and vegetables, unsaturated fats, vitamin D and whole grains were associated with better mental health among immigrants in Western societies, partly through improved self-esteem and physical activity^(16)^. In contrast, acculturation to energy-dense Western diets and experiences of food insecurity were linked to poorer mental health, including depression and anxiety, driven by factors such as non-communicable diseases, family conflict, stigma and inability to afford culturally appropriate foods. Conversely, consumption of ethnic foods was found to support immigrants’ mental well-being^(16)^.

However, not all immigrants are the same and heterogeneity within migrant populations to high-income countries exist in terms of health outcomes, dietary behaviours, the country or region of origin, ethnicity, soci-oeconomic status, sex, age at arrival and the specific environmental and climate/weather context of the host country. In particular, would immigration from hotter/sunnier towards colder less sunny climates make people put on weight upon arrival in the destination country possibly due to changes in dietary habits, physical activity levels and physiological and/or psychological responses to temperature change^(17)^. Literature has shown that exposure to cold temperatures induces behavioural and physiological changes that generate or conserve heat. For example, short-term exposure to cold temperatures increases food intake and metabolic energy expenditure, while long-term exposure to cold can lead to the accumulation of insulating fat and a larger, rounder body shape that reduces heat loss by increasing the ratio of body volume to surface area^(18–20)^.

Besides, newly arriving immigrants, whether as students, refugees or employees, experience to a varying extent some sort of stress and anxiety that comes with moving into a new country and this may impact their well-being and lead to unhealthy eating habits, which contribute to weight gain and obesity^(15,21)^. Immigrants during their first years in the destination country have to face changing social, economic, cultural and climatic factors which may impact their eating habits, physical activity, physiological health (e.g. hormones fluctuations, change in gut microbiome) and also put pressure on their mental health and well-being^(21)^. All of this might collectively contribute to weight gain and obesity among immigrants compared with the population born in the host country.

Given the complexity and inconclusive evidence pertaining this topic, further research is needed to reveal the underlying socio-economic, cultural and environmental multifactorial mechanisms behind weight gain and obesity among immigrants to high-income countries over time. Accordingly, this study aims to conduct an umbrella literature review on the impacts of cultural, economic and climate variations on obesity and weight gain amongst immigrants to high-income countries. The umbrella literature review is a method that collates evidence from multiple systematic reviews, which would provide a holistic understanding of the factors contributing to obesity and weight gain in immigrant populations and identify areas for future research and policy intervention. Through this synthesis, we aim to inform public health strategies and contribute to the development of targeted interventions that promote health equity for immigrant populations in the long term.

## Methods

### Study design

This is an umbrella review of the literature, synthesising evidence from existing systematic reviews to provide a comprehensive overview of the impacts of socio-economic, cultural and environmental factors on obesity and overweight among immigrants to high-income countries. Umbrella literature reviews are particularly suited for summarising broad and complex topics by integrating findings across multiple reviews, thereby offering a higher-level synthesis of evidence^(22)^.

### Literature search and eligibility criteria

A systematic search was conducted in PubMed, Web of Science and Scopus databases using a combination of keywords and Boolean operators from January 2015 to November 2025 for reviews and systematic reviews (with or without meta-analyses) of observational studies published in English. These three databases were chosen as they cover the medical field and other fields including the Science, Technology, Engineering and Mathematics and social sciences where most of the reviews on the topic of interest could be captured. The time period between 2015 and 2025 (i.e. reviews published in the past 10 years) was chosen so that the focus would be on what is happening recently with respect to obesity and immigration in an era of conflicts and global climate and socio-economic issues triggering large immigration waves to provide an up-to-date robust evidence for policymaking and community initiatives.

The search strategy was designed to capture literature that addressed the intersection of migration (including all types of immigrants – economic migrants, international students and refugees), obesity, socio-economic and environmental determinants (i.e. cultural and physical environmental factors related to climate/weather). The search terms focused on the following keywords: (1) immigrants, (2) migrants, (3) refugees, (4) weight gain, (5) obesity, (6) climate, (7) culture, (8) migrating population, (9) environment, (10) acculturation, (11) dietary changes, (12) lifestyle changes, (13) social environment, (14) food preferences, (15) tradition, (16) socio-economic factors, (17) poverty, (18) income, (19) employment, (20) food security, (21) physical environment, (22) physical activity, (23) sedentary behaviour, (24) green spaces and (25) high-income countries. The search algorithms used can be found in online Supplementary Table 1.

### Inclusion and exclusion criteria

Only reviews and systematic reviews with or without meta-analyses were included. We also focused only on immigrants to high-income countries. We included all types of immigrants – economic migrants, international students and refugees/asylum seekers. The inclusion criteria were composed of articles addressing obesity and/or weight gain as a primary or secondary outcome and that discuss cultural, socio-economic and/or environmental factors. On the other hand, we excluded studies focusing exclusively on children or adolescents. In other words, only articles focusing on/include adult population were included in this umbrella review. Reviews and systematic reviews not involving immigrants to high-income countries and focusing on other non-communicable diseases such as CVD and diabetes with no direct reference to obesity and weight gain were also excluded. Additionally, we excluded non-English language publications, narrative reviews that do not include a systematic search, reviews of only qualitative studies, editorials, conference proceedings, commentaries and opinion pieces reviews. Finally, reviews focusing on obesity public health interventions were also excluded because they do not align with the aims of this umbrella review, which is to summarise the impacts of cultural, socio-economic and climate variations on obesity and weight gain among immigrants.

### Screening and Selection Process

The initial search across all search algorithms yielded a total of 363, 581 and 349 articles in PubMed, Web of Science and Scopus, respectively (online Supplementary Table 1). These articles were exported to Endnote software for duplicates removal, screening and data extraction. We removed 384 articles as they were duplicates between the three search databases, leaving a total of 909 articles that underwent title and abstract independent double screening (once by researcher MAAA and once by researcher MF) following the pre-identified inclusion and exclusion criteria. Both researchers agreed to exclude 827 articles after the title and abstract screening phase. This left eighty-two articles that underwent full-text screening. Of the eighty-two articles screened, forty-eight were excluded because they were non-systematic narrative reviews, focused only on qualitative evidence, examined obesity interventions rather than determinants, targeted children or youth, addressed other non-communicable diseases without a direct focus on obesity or weight gain, did not specifically study immigrants from low- and middle-income to high-income countries or were conference proceedings or commentaries. One additional study (Shad *et al.*, 2024) was excluded as it focused on post-migration microbiome changes rather than obesity or weight change^(23)^. As a result, data extraction and synthesis were performed on the remaining thirty-three systematic review articles.

### Data extraction and synthesis

Data were extracted from each of the included thirty-three reviews focusing on the article’s objectives, the databases searched, the time period covered, the types of articles reviewed, the tools/methods of measurement used in the reviewed articles, the effect sizes of the reviewed studies, the characteristics of the studied immigrant populations and the key findings related to cultural, socio-economic and physical climate factors impacting obesity and weight gain amongst immigrants to high-income countries. Additionally, thematic synthesis for the factors impacting obesity and weight gain among immigrants to high-income countries was used to categorise the findings of the thirty-three included reviews under three main subheadings:

1. Physical climate and metabolic/physiological changes.
2. Socio-economic stressors and psychological mediators.
3. Cultural factors

The thematic synthesis allows for a comprehensive and organised presentation of the evidence, highlighting both commonalities and divergences across immigrant populations and host country contexts. These three themes were deduced from the thirty-three included systematic reviews and from other observational quantitative and qualitative studies on the topic of interest as emerging themes that are repeated across the studies.

Finally, methodological quality and strength of the thirty-thee review articles were evaluated using the ‘A Measurement Tool to Assess Systematic Reviews’ tool. This tool relies on eleven criterion items to measure the methodological quality of systematic reviews and their meta-analysis. If the specific criterion is met, one point is allocated. An overall score relating to review quality is then calculated using the sum of the individual scores. A review scoring 8 and above is considered high quality, 4–7 is a review of moderate quality and below 4 is low quality^(24–26)^.

### Patient involvement

No patients were involved in setting the research question or the outcome measures, nor were they involved in developing plans for design or implementation of the study. No patients were asked to advise on interpretation or writing up of results. It was not evaluated whether the studies included in the review had any patient involvement. The results will be disseminated to the general public through public presentations and authors’ involvement in different community and academic events.

## Results

In total, thirty-three systematic review articles were included in the synthesis of this umbrella literature review. The key findings and thematic classification of the thirty-three review articles are summarised in Table 1. Additionally, we included a more detailed systematic summary of the characteristics of the thirty-three review articles in Table 2.

**Table 1.** A table summarising narratively the thirty-three review articles included in the synthesis of this umbrella literature review Table 1 long description.

Review article	Review type	Thematic subheading	Summary of key findings
Fernando *et al.*, 2015^(31)^	Systematic Literature review. No meta-analysis	Physical climate and metabolic/physiological changes. Socio-economic stressors and psychological mediators. Cultural factors.	This review article is about South Asian migrants (SA) to high-income countries who experience a disproportionately high burden of CVD, particularly coronary artery disease (CAD), with prevalence rates 1·5–2 times higher than Europids (i.e. whites of European ancestry). SA tend to present with CAD 6–10 years earlier and exhibit higher rates of diabetes mellitus (DM), insulin resistance, visceral adiposity and dyslipidaemia, which are primary drivers of this excess risk. At any given BMI, SA have greater body fat and metabolic abnormalities, contributing to early onset of disease. Lifestyle changes post-migration, including reduced physical activity and adoption of Western dietary patterns, further exacerbate risk. Evidence on prognosis is mixed: while some studies report higher mortality after acute coronary syndromes, others suggest similar or better long-term outcomes compared to Europids, possibly due to preserved cardiac function and lower atrial fibrillation rates. Stroke and heart failure data are limited, but SA appear to present younger and with higher DM prevalence. Emerging research highlights roles for adipokines, genetic predisposition and early life factors in shaping risk. Overall, culturally tailored interventions targeting obesity, DM, diet and physical activity, alongside improved medication adherence, are critical to mitigating CVD burden in this vulnerable population.
Gasevic *et al.*, 2015^(59)^	Systematic literature review. No meta-analysis	Physical climate and metabolic/physiological changes. Socio-economic stressors and psychological mediators. Cultural factors.	This systematic review synthesised evidence from 110 studies published between 2000 and 2014 on ethnic differences in modifiable CVD risk factors in North America. Most studies (86 %) were U.S.-based, with the remainder from Canada, and nearly 90 % were cross-sectional. The review compared six major ethnic groups – Indigenous, Black, Chinese, Hispanic, Filipino and Arab – against White populations. Findings consistently showed higher hypertension prevalence among Black individuals and higher diabetes, overall and abdominal obesity and smoking rates among Indigenous populations compared with Whites. Hispanic individuals exhibited greater diabetes prevalence, while Filipino populations demonstrated higher rates of hypertension and diabetes. Conversely, Chinese and Filipino individuals generally had lower overall obesity and smoking prevalence than Whites. Evidence for Arab, Chinese and Filipino groups was limited and sometimes inconsistent. The review also noted that many studies lacked adjustment for confounders and relied on self-reported data, contributing to variability in findings. Potential explanations for disparities include differences in body fat distribution, socio-economic conditions, cultural attitudes towards weight, health behaviours and stress exposure. The authors call for more robust longitudinal research and culturally tailored interventions to address ethnic-specific risk profiles and reduce CVD disparities.
Goulão *et al.*, 2015^(48)^	Systematic literature review. No meta-analysis	Socio-economic stressors and psychological mediators. Cultural factors.	This review summarises knowledge regarding the impact of immigration on BMI. The findings indicate that the effect of migration on BMI varies depending on the immigrants’ ethnic background, country of origin and the host country. A consistent positive association between increased BMI and longer time since immigration was observed among Hispanic, European and African immigrants. For Asian immigrants, the results were less consistent, with fewer than half the studies showing a positive association, and some indicating an inverse relationship or no significant link. Generally, immigrants tend to have a lower BMI upon arrival compared with the population born in the host country, but this health advantage often diminishes over time. Factors contributing to this weight gain post-migration include changes in dietary habits (often adopting diets higher in fat and sugar and lower in fiber), reduced physical activity levels and potentially acculturation stress. They conclude that immigration appears to have a detrimental effect on BMI over time for many groups, warranting further research with improved methodologies to better understand the causal factors and develop effective public health interventions.
Kershaw & Albrecht, 2015^(44)^	Systematic literature review. No meta-analysis	Socio-economic stressors and psychological mediators. Cultural factors.	This review synthesises United States studies examining racial/ethnic residential segregation and cardiovascular risk, with emphasis on obesity and weight-related outcomes among non-Hispanic Blacks and Hispanics. Most studies were cross-sectional, using metropolitan-level isolation indices or neighbourhood racial composition. Findings for Blacks consistently linked higher segregation to increased BMI and obesity prevalence, particularly among women, and in some cases, persistent exposure predicted weight gain over time. For Hispanics, results were mixed: metropolitan-level segregation was associated with lower obesity among Mexican-American women but higher obesity among Hispanic White women, while neighbourhood-level studies often reported null or positive associations. These patterns reflect complex pathways involving physical/metabolic factors (biological risk markers like BMI and hypertension), socio-economic stressors (poverty concentration, limited access to health-promoting resources and neighbourhood disadvantage) and cultural influences (immigrant enclaves offering dietary continuity and social support, but also barriers to physical activity and healthcare). While Hispanic enclaves sometimes promoted healthier diets, they also correlated with high poverty and reduced treatment uptake for hypertension. The review underscores the need for prospective studies and culturally informed interventions addressing structural inequities and acculturation processes that shape obesity risk in segregated communities.
Gong & Zhao, 2016^(46)^	Systematic literature review. No meta-analysis	Physical climate and metabolic/physiological changes. Socio-economic stressors and psychological mediators. Cultural factors.	This systematic review of sixteen studies examines CVD and risk factors among Chinese immigrants compared with mainland Chinese. Migration significantly alters epidemiological patterns: stroke prevalence and mortality decline sharply post-migration, while CHD prevalence rises from 0·8 % in China to 1·7–3·2 % in Canada/UK and up to 24 % in Mauritius. Obesity and related metabolic risks increase with acculturation; although Chinese immigrants maintain lower BMI than Whites, their obesity prevalence surpasses mainland Chinese and escalates with longer residence. Dietary acculturation – marked by higher intake of fats, sugars and processed foods – combined with reduced physical activity drives this trend. Diabetes prevalence among immigrants (12–13 % in USA) doubles that of mainland China (5·5 %), and hypertension prevalence reaches 35–54 %, with odds rising by 26 % after 30 years in the USA. These findings reflect three themes: metabolic/physiological changes (higher cholesterol, BMI-linked risk), socio-economic stressors (urbanisation, healthcare gaps and acculturation stress) and cultural factors (loss of traditional diets, adoption of Western habits). Targeted interventions addressing lifestyle and cultural adaptation are critical to mitigate obesity and CVD risk in Chinese immigrant populations.
Wang *et al.*, 2016^(38)^	Systematic literature review. No meta-analysis	Physical climate and metabolic/physiological changes. Socio-economic stressors and psychological mediators. Cultural factors.	This systematic review of eighteen studies examines dietary changes and adaptation among refugees resettled in the USA. Findings reveal a consistent pattern of increased energy intake post-resettlement, driven by higher consumption of meat, eggs, high-fat foods, sweets, fast food and sugar-sweetened beverages. While some groups increased fruit, vegetable and dairy intake, others reported declines, often linked to food insecurity and low acculturation. Overweight and obesity emerged as growing concerns, particularly among refugees with histories of food deprivation; Cambodian women with higher deprivation scores were more likely to consume fatty meats and be overweight (OR: 1·2). Somali households facing child hunger showed extreme odds for daily egg (OR: 21·2) and meat intake (OR: 11·2). Acculturation and obesity were linked to metabolic/physiological factors such as nutrient and vitamin deficiency (e.g. Vitamin B_12_ deficiency), socio-economic stressors (poverty, language barriers and depression) and cultural factors (retention of traditional diets, discomfort with processed foods and unfamiliarity with United States grocery systems). Acculturation influences were evident: longer residence in the USA correlated with greater intake of processed foods and added sugars, while adolescents adopted American fast-food habits. Refugees expressed a need for culturally tailored nutrition education and cooking classes. These findings underscore the urgency for interventions addressing obesity risk and promoting healthy adaptation in refugee populations.
Murphy *et al.*, 2017^(10)^	Systematic literature review. No meta-analysis	Physical climate and metabolic/physiological changes. Socio-economic stressors and psychological mediators. Cultural factors.	This review explores obesity risk among international migrants, particularly those moving from low- and middle-income countries to high-income countries. Migrants often arrive with a health advantage, including lower BMI, due to the ‘healthy migrant effect’ and earlier stages of the nutrition transition in source countries. However, obesity prevalence rises sharply after 10–15 years post-migration, frequently surpassing domestic populations. Key drivers include metabolic/physiological factors (genetic predisposition, epigenetic programming from early undernutrition), socio-economic stressors (poverty, marginalisation, racism and stress-related allostatic load) and cultural influences (dietary acculturation, body size norms and barriers to leisure-time physical activity). While migrants often retain healthier cooking habits and consume more fruits and vegetables than natives, adoption of processed foods and fast-food increases obesity risk. Physical activity declines as occupational and travel-related activity is replaced by sedentary lifestyles, compounded by cultural and environmental barriers. Acculturation generally predicts higher obesity risk, particularly in men. The review underscores the need for culturally tailored interventions, as evidence suggests these approaches improve effectiveness. Addressing obesity in migrant populations requires integrating cultural sensitivity with strategies to mitigate socio-economic disadvantage and stress.
Alidu & Grunfeld, 2018^(47)^	Systematic literature review. No meta-analysis	Cultural factors	This review aimed to identify associations between acculturation and body weight among immigrants to high-income countries. It was found that higher acculturation levels were generally linked to increased BMI or obesity, though some exceptions existed. Longer duration of residence in the host country was also commonly associated with higher obesity rates, particularly among first-generation migrants. Second-generation migrants often exhibited even higher obesity rates and more unhealthy behaviours than first-generation counterparts. Language proficiency in the host country was associated with lower obesity rates, while social acculturation showed mixed results. Overall, acculturation, duration of residence and generational status significantly influenced obesity risk, often mediated by changes in diet and physical activity.
Ngongalah *et al.*, 2018^(35)^	Systematic literature review. No meta-analysis	Physical climate and metabolic/physiological changes. Socio-economic stressors and psychological mediators. Cultural factors.	This article synthesises evidence on the dietary and physical activity behaviours and their determinants, among women of childbearing age, including pregnant women, who migrated from African countries to live in high-income countries (HIC). Findings suggest potential dietary inadequacies in micronutrients such as Fe, folate and Ca, alongside excessive Na intake. Dietary patterns were generally bicultural, indicating women retained some traditional African dietary practices while also adopting Western-style habits such as increased consumption of snacks, processed foods and eating out more frequently. No women were found to have completely abandoned their traditional diets. Evidence regarding physical activity levels was conflicting, possibly due to variations in assessment methods and populations studied. Key determinants influencing both diet and physical activity included the post-migration environment (e.g. food availability, weather and work schedules), cultural and religious beliefs (particularly among Muslim women), pregnancy status, personal beliefs about food and exercise and competing priorities like work and family care. The review concludes that acculturation impacts the health behaviours of African migrant women in complex ways.
Mansour *et al.*, 2020^(37)^	Systematic literature review. No meta-analysis	Socioe-conomic stressors and psychological mediators. Cultural factors.	This systematic review examined food insecurity among Middle Eastern and North African (MENA) migrants and refugees in high-income countries. Three US-based studies revealed alarmingly high food insecurity rates (40–71 %), far exceeding general population levels. Determinants included recent arrival, low English proficiency, limited education and strong cultural preferences for traditional foods. Food insecurity was linked to adverse health outcomes, illustrating a paradox of both undernutrition and overweight. Among Somali women, food-insecure participants were nearly three times more likely to be overweight or obese (OR: 2·66). Sudanese children exhibited complex risks: 26·6 % were overweight/obese by BMI, yet 38 % had low spinal bone density. These findings underscore the interplay of metabolic vulnerabilities, socio-economic stressors (poverty, language barriers) and cultural factors (traditional food retention, religious dietary norms). Food insecurity among MENA migrants poses long-term risks for chronic disease, osteoporosis, obesity and cardiovascular complications. The review calls for culturally tailored interventions, longitudinal research and improved measurement tools to address this critical public health issue.
Amiri, 2021^(2)^	Systematic literature review and meta-analysis	Socio-economic stressors and psychological mediators. Cultural factors.	This meta-analysis provides the first global pooled estimates of obesity and overweight among immigrants and refugees. Across fifty-nine studies, nearly half of the immigrant population had BMI ≥ 25, with obesity prevalence at 24 % and overweight at 37 %. Sex differences were evident: women had higher obesity rates (27 %) than men (20 %), while men were more likely to be overweight (44 % *v*. 34 %). Immigrants and refugees showed similar obesity prevalence (24 % *v*. 23 %), but overweight was more common among immigrants (38 % *v*. 29 %). Regional disparities emerged, with obesity highest in the Americas (28 %) and lowest in Australia (20 %). Measured BMI yielded slightly higher prevalence than self-reported data. The review highlights that migration to high-income countries often coincides with lifestyle changes, including increased consumption of energy-dense foods and reduced physical activity, contributing to weight gain. Cultural factors, such as dietary acculturation and regional norms, influence these patterns. Socio-economic stressors further exacerbate obesity risk. Given obesity’s association with diabetes, CVD and mental health issues, these findings underscore the need for culturally tailored interventions and longitudinal research to monitor weight trajectories among migrant populations.
Bains *et al.*, 2021^(33)^	Systematic literature review. No meta-analysis	Physical climate and metabolic/physiological changes. Socio-economic stressors and psychological mediators. Cultural factors.	This exploratory review synthesises evidence from forty-four studies on prenatal health among immigrant women in Norway. Gestational diabetes prevalence was markedly higher among women from Asia and Africa, with South Asian women exhibiting greater insulin resistance and lower beta-cell function. Weight-related disparities included higher gestational weight gain among women from Africa and the Middle East and increased abdominal obesity among South Asians, compounded by lower physical activity levels. Micronutrient deficiencies were striking: severe vitamin D deficiency affected up to 45 % of South Asian women, and folate supplement use was significantly lower among immigrants, though improved with longer residence. Cultural and socio-economic factors – such as language barriers, incongruent dietary advice and limited health literacy – shaped health behaviours and service experiences. The review underscores the need for culturally tailored interventions, improved screening and qualitative research to address gaps for vulnerable groups, including refugees and undocumented immigrants.
Ankomah *et al.*, 2022^(60)^	Systematic literature review. No meta-analysis	Physical climate and metabolic/physiological changes. Socio-economic stressors and psychological mediators. Cultural factors.	This systematic review synthesises evidence on the double burden of malnutrition (DBM) among migrants and refugees in high-income countries. Across five studies, DBM manifested as the coexistence of undernutrition (stunting, wasting and underweight) and overnutrition (overweight, obesity) within the same populations. Overnutrition prevalence ranged from 11·1 % to 42 %, while undernutrition ranged from 0·3 % to 17 %. For example, adult immigrants in Geneva had underweight (5·7 %) and overweight (42 %). These patterns reflect a transition from early-life deprivation to obesogenic environments post-migration, driven by acculturation, socio-economic stressors and food system changes. Migrants often face barriers to using local produce and rely on affordable ultra-processed foods, increasing obesity risk. Cultural factors, such as unfamiliarity with host-country diets, compound these challenges. The review underscores the urgent need for integrated interventions addressing both undernutrition and obesity, alongside policies targeting food environments and culturally tailored nutrition education. Evidence gaps persist, particularly regarding longitudinal impacts and qualitative insights into lived experiences.
Berggreen-Clausen *et al.*, 2022^(36)^	Systematic scoping literature review. No meta-analysis	Physical climate and metabolic/physiological changes. Socio-economic stressors and psychological mediators. Cultural factors.	This scoping review maps and characterises how immigrants from low- and middle-income countries interact with the food environments in high-income host countries. Key findings show that immigrants strongly value and prefer fresh, natural, high-quality, traditional foods, perceiving them as healthier than processed host-country options. However, they face numerous barriers in accessing these foods. Physically, their neighbourhoods often have limited availability of affordable, good-quality fresh produce and cultural items, alongside high access to unhealthy options. Weather conditions and cold season were also an added challenge when relying on public transport to access food. Economically, low incomes and the high cost of preferred foods create significant constraints, leading to food insecurity and reliance on food assistance programs, which themselves can be difficult to navigate or insufficient. Socio-culturally, while adults often maintain preferences for traditional diets, children are influenced by the host country’s food environment (e.g. schools) and frequently demand processed or fast foods, creating household conflict The review concludes that immigrants actively strive to maintain healthier, traditional diets but are often forced to make compromises due to structural and family-level barriers, highlighting the need for policies and interventions that address these complex interactions to support healthier eating among vulnerable populations.
Chapagai & Fink 2022^(61)^	Systematic Literature review. No meta-analysis	Physical climate and metabolic/physiological changes. Socio-economic stressors and psychological mediators. Cultural factors.	This scoping review synthesises evidence on sleep disorders and cardiovascular risk in South Asians, including migrants in the USA, UK, Canada and the Netherlands. Obesity emerges as a critical factor across studies, strongly associated with obstructive sleep apnea (OSA) and cardiometabolic risk. Migrant South Asians often exhibit higher BMI, increased body fat and elevated hemoglobin A1C compared with European counterparts, highlighting weight gain as a key health concern. Physiological markers – BMI, lipid profiles and glucose – were central to understanding obesity-related risk. Immigration-related stress was linked to poor sleep, which may exacerbate metabolic dysfunction, though psychological pathways were not deeply explored. Acculturation influenced dietary patterns, with traditional practices favouring high-fat foods and assimilation linked to animal protein diets. These cultural shifts, combined with lifestyle changes in host countries, contribute to obesity and related cardiovascular risks. Overall, the review underscores the interplay of cultural adaptation, socio-economic context and physiological vulnerability in shaping obesity among South Asian immigrants, while calling for research that integrates stress and cultural determinants into preventive strategies.
Ismail *et al.*, 2022^(32)^	Systematic literature review. No meta-analysis	Physical climate and metabolic/physiological changes. Socio-economic stressors and psychological mediators. Cultural factors.	This systematic review of 10 studies examines how socioeconomic determinants shape CVD, obesity and diabetes among UK migrants. Obesity and weight gain feature prominently: Ghanaian migrants in London show dramatically higher obesity (men PR 15·04; women PR 6·63) and abdominal obesity (men PR 10·48; women PR 2·56) than rural Ghanaian counterparts, and adult waist circumference rises with area deprivation, especially in men. Childhood overweight and BMI trajectories also vary by socio-economic position and maternal nativity/ethnicity, indicating early‑life divergence in weight gain patterns. Metabolic/physiological changes are central through BMI and waist circumference indicators and elevated calculated CVD risk among migrants. Socio-economic stressors including education, income, occupational class and neighbourhood deprivation are consistently implicated in higher adiposity, operating through food affordability, access to healthy options and activity resources. Cultural factors emerge via English language proficiency – linked to lower undetected hypertension and diabetes in some South Asian subgroups – alongside ethnic heterogeneity suggesting different patterns of dietary acculturation and health system navigation. Collectively, the review underscores that obesity and related cardiometabolic risks in UK migrants reflect intertwined socio-economic disadvantage, cultural adaptation and physiological vulnerability, warranting targeted, context‑specific prevention strategies.
Matsangos *et al.*, 2022^(34)^	Systematic Literature review. No meta-analysis	Physical climate and metabolic/physiological changes. Socio-economic stressors and psychological mediators. Cultural factors.	This review examines Afghan refugees’ health status in Europe, highlighting obesity and related non-communicable diseases (NCD) as major concerns. Reported prevalence of overweight ranges from 32 to 38 %, obesity from 31 %, hypertension 46 % and diabetes 13 %, alongside widespread dyslipidaemia and tobacco use. These patterns reflect profound metabolic changes post-migration, compounded by malnutrition and vitamin D deficiency. Reduced sun exposure and poor diet are linked to NCD risk. Socioeconomic stressors – poverty, food insecurity and inadequate healthcare – intersect with psychological mediators like depression, which are highly prevalent and associated with obesity and CVD. Cultural factors further shape health outcomes: gender inequity and health illiteracy limit Afghan women’s access to antenatal and preventive care, while cultural stigma around mental health and sexuality impedes help-seeking. Traditional dietary patterns and low physical activity exacerbate weight gain in resettlement contexts. Overall, the review underscores the need for integrated strategies addressing socio-economic disadvantage, cultural barriers and trauma-informed care to mitigate obesity and cardiometabolic risk among Afghan refugees in Europe.
Mensah *et al.*, 2022^(42)^	Systematic literature review and meta-analysis	Socio-economic stressors and psychological mediators.	This systematic review examines the prevalence of cardiometabolic risk factors among sub-Saharan African (SSA) immigrants living in high-income countries. The review found considerable heterogeneity in the burden of risk factors depending on the immigrants’ country of origin and their host country. Gender differences were noted, with overweight/obesity generally higher among women, while hypertension, diabetes, dyslipidaemia (mixed findings) and smoking were often higher among men. The authors conclude that SSA immigrants face a significant burden of cardiometabolic risk factors in high-income countries, underscoring the need for targeted preventive health strategies, policies to improve healthcare access and interventions to advance health equity for this population.
Osei *et al.*, 2022^(43)^	Systematic literature review. No meta-analysis	Socio-economic stressors and psychological mediators. Cultural factors.	This review maps aetiological research on migrants in Germany and shows that adiposity and cardiometabolic risk are recurrent outcomes, often studied alongside mental health and health behaviours. Socio-economic stressors (education, occupation and affluence) consistently relate to obesity and non-communicable diseases (NCD), with lower status linked to adverse outcomes and unhealthy behaviours, while some cardiometabolic associations remain complex due to lifestyle mediation. Psychological mediators – including discrimination, acculturation stress and distress – are repeatedly examined and correlate with both mental health and lifestyle patterns that influence weight gain. Cultural factors (acculturation, language skills and traditions/norms) shape healthcare use, diet and smoking, contributing to heterogeneity in obesity risk across migrant groups. The review also highlights BMI, insulin resistance and early‑life exposures as mechanisms underlying obesity in migrants, especially first‑generation individuals exposed to different nutritional environments prenatally and in childhood. Overall, the evidence suggests interacting social, cultural and biological pathways to weight gain, but meta‑analytical effect sizes are lacking due to heterogeneity; the authors call for longitudinal and experimental studies to quantify magnitudes and clarify causal links.
LeCroy *et al.*, 2023^(41)^	Systematic literature review. No meta-analysis	Socio-economic stressors and psychological mediators. Cultural factors.	This review discusses immigration as a structural determinant of health (SDH), shifting the focus beyond individual-level dietary acculturation to encompass broader systemic factors that influence immigrant diet and cardiometabolic health. The authors reviewed quantitative, qualitative and mixed-methods studies, observing that while cardiometabolic risks vary by country of origin, immigrants often share common experiences shaped by structural barriers upon resettlement. These shared SDH include challenges within the neighbourhood food environment (e.g. limited access to affordable, healthy, culturally familiar foods), economic instability (e.g. poverty limiting food choices, reliance on potentially inadequate food assistance), barriers to healthcare access and quality, detrimental social contexts (e.g. discrimination, acculturative stress) and difficulties accessing culturally and linguistically appropriate education, including nutrition information. The review suggests that these structural factors, often linked to immigration policies and societal inequities, create significant obstacles to maintaining healthy dietary patterns and contribute to cardiometabolic disease disparities across diverse immigrant groups.
Lopez *et al.*, 2023^(51)^	Systematic literature review and meta-analysis	Physical climate and metabolic/physiological changes. Socio-economic stressors and psychological mediators. Cultural factors.	This systematic review/meta‑analysis examines whether developmental mismatches between constrained prenatal environments and postnatal obesogenic contexts raise obesity and cardiometabolic risks. Across 38 studies, migrants had higher type 2 diabetes (T2D) prevalence than hosts (PR 1·48) and origins (PR 1·80), while adult obesity was not higher *v*. hosts but greater than origins, and child obesity was elevated (PR 1·22), especially among Asian migrant children. These patterns align with physiological mechanisms – developmental plasticity and epigenetic processes – that can backfire when predicted environments diverge from actual ones. Socio-economic factors are woven into design and interpretation: studies controlled for age, sex, SES/education and the discussion situates modern LMIC‑to‑HIC migration within rapid mortality declines and changing living conditions, with maternal stress/nutritional signals shaping fetal programming. Cultural dynamics – acculturation/assimilation and selection – help explain destination‑specific differences (e.g. African migrants’ obesity excess in Europe, not North America), indicating that post‑migration norms and trajectories modulate weight gain beyond biology alone. Overall, the review supports links between early‑life developmental tuning and later obesity/weight‑related outcomes, while acknowledging that socio-economic and cultural processes shape how these physiological susceptibilities are expressed across migrant contexts.
Paixão *et al.*, 2023^(28)^	Systematic literature review and meta-analysis	Socio-economic stressors and psychological mediators. Cultural factors.	This study systematically reviewed and performed a meta-analysis to determine the prevalence of metabolic syndrome and its individual components among Latino immigrants living in the USA. The pooled prevalence of metabolic syndrome was found to be 33·1 %, indicating that approximately one-third of Latino immigrants in the USA meet the criteria for this condition. Among the individual components, low HDL-cholesterol (46·0 %) and high waist circumference (44·8 %) were the most prevalent, followed by high blood pressure (33·9 %), high TAG (32·6 %) and high fasting glucose (26·1 %). The authors noted significant heterogeneity between studies, likely attributable to differences in diagnostic criteria, study populations (including country of origin), socio-economic status and length of residence in the USA. Several reviewed studies suggested that the risk of metabolic syndrome increases with longer duration of US residence, potentially linked to acculturation-related changes in diet and physical activity, as well as stress. The review concludes that metabolic syndrome represents a significant public health concern for Latino immigrants in the USA, elevating their risk for CVD and type 2 diabetes.
Ufholz & Werner 2023^(40)^	Systematic Literature review. No meta-analysis	Socio-economic stressors and psychological mediators. Cultural factors.	This narrative review synthesises 2018–2023 evidence on who eats fast food (FF) and how that relates to obesity risk. Across U.S. data (NHANES) and international studies, FF use is common and highest among younger adults, men and Black Americans; patterns often follow an inverse‑U for income, with middle‑income groups consuming more than the poorest or wealthiest. Employment and longer work hours correlate with higher FF intake, underscoring time constraints as a socioeconomic driver. Frequent FF intake (≥ 2–3 × /week) predicts BMI gain, greater visceral and liver fat. Cultural factors shape exposure and choices: immigrant Latinos – particularly undocumented women – report less FF than U.S.‑born Latinos, while cross‑national data suggest norms influence frequency and preferred FF items. Psychological mediators are implied (time pressure, possibly discrimination) but not consistently modelled. Overall, the review supports susceptibility to weight gain from energy‑dense FF, socio-economic pressures that channel people toward convenience and cultural contexts that condition who eats FF and how often – together contributing to obesity and cardiometabolic disparities
Kibibi *et al.*, 2024^(30)^	Systematic literature review and meta-analysis	Physical climate and metabolic/physiological changes. Socio-economic stressors and psychological mediators. Cultural factors.	This meta-analysis examined whether refugees resettled in high-income countries face higher obesity risk compared to host populations. Across nine observational studies (United States, Europe and Asia-Pacific), pooled results showed no significant overall difference (OR = 1·11; 95 % CI: 0·56, 2·20), though heterogeneity was high. Subgroup analysis revealed refugee men had significantly lower odds of obesity (OR = 0·72), while women showed a non-significant trend towards higher risk. Physiological pathways – BMI increase, metabolic syndrome and cardiometabolic disease – are discussed as consequences of dietary and lifestyle changes post-resettlement. Socio-economic stressors, including food insecurity, limited income and urban living, intersect with cultural adaptation and acculturation, shaping exposure to obesogenic environments. Psychological stress from displacement and integration challenges may compound these risks. Cultural factors such as ethnic origin and pre-resettlement living conditions (camp *v*. urban) influence vulnerability, with evidence that Sub-Saharan African refugees experience rapid weight gain over time. While physical climate was not addressed, structural determinants – access to affordable healthy food and opportunities for physical activity – emerge as critical. Findings underscore the need for culturally tailored nutrition interventions and systemic changes to mitigate obesity risk among refugees, promoting equitable health outcomes post-resettlement.
Nayak *et al.*, 2024^(62)^	Systematic literature review. No meta-analysis	Socio-economic stressors and psychological mediators. Cultural factors.	This scoping review synthesised 13 studies on the health of undocumented Asian immigrants (UAI) in the USA, a rapidly growing yet understudied population. Research remains sparse and concentrated in California, with eight studies using BRAVE datasets. Health topics included mental health (depression, psychosocial needs), self-rated health, chronic conditions (diabetes, hypertension and obesity), healthcare access barriers, COVID-19 outcomes, contraceptive use and HIV. Findings highlight structural vulnerabilities: undocumented status intersects with socio-economic stressors – loss of insurance, employment instability and immigration enforcement – while cultural and racialised factors such as Islamophobia and anti-Asian xenophobia compound health risks. Psychological mediators, including fear and stress, were linked to poor mental health and delayed care. Chronic disease prevalence and obesity were examined in national datasets, but evidence is inconsistent and limited by small sample sizes and lack of disaggregation by ethnicity. The review calls for robust, diverse samples, granular data and intersectional frameworks to capture heterogeneity within UAI. Overall, this emerging field underscores the urgent need for culturally tailored interventions and policy reforms to reduce health inequities among UAI
Purcino & Bedrikow, 2024^(39)^	Systematic scoping literature review. No meta-analysis	Socio-economic stressors and psychological mediators. Cultural factors.	This review aimed to map and describe the existing evidence regarding the dietary characteristics, nutrition and diet-related health conditions of Haitian immigrants across the globe. The review highlights significant dietary changes Haitian immigrants experience post-migration, often characterised by a shift away from traditional diets rich in fruits, vegetables and legumes towards increased consumption of processed foods, sugary drinks and fast food. Factors driving these changes include the food environment of the host country (availability, accessibility and affordability), socio-economic constraints (low income, high cost of healthy foods), limited time for food preparation due to work demands, cultural adaptation challenges and sometimes a lack of nutritional knowledge. Food insecurity emerged as a major threat, linked to poverty, unstable employment, language barriers and difficulties accessing food aid. Concurrently, diet-related health issues such as overweight, obesity, hypertension and diabetes are noted concerns within this population.
Vo *et al.*, 2024^(29)^	Systematic literature review. No meta-analysis	Physical climate and metabolic/physiological changes. Socio-economic stressors and psychological mediators. Cultural factors.	This narrative review examines how acculturation influences cardiovascular risk among Asian American immigrants, particularly Southeast Asians. Using evidence from NHANES, NHIS, MASALA and MESA cohorts, the review highlights that proxies such as English spoken at home, length of stay, religiosity and admixed family structures modulate cardiometabolic health. Longer United States residency correlates with higher odds of diabetes (OR = 1·5) and prediabetes (OR = 1·3), supporting the ‘negative acculturation effect.’ Language use shows mixed associations: greater English use linked to higher hypertension prevalence but lower smoking rates among those maintaining native language. Religiosity, surprisingly, was associated with higher systolic BP and BMI in MESA, while MASALA found assimilation linked to lower obesity and diabetes among South Asian women, alongside healthier behaviours (more exercise, fewer calories). Cultural adaptation, dietary shifts towards high-fat Western diets and psychosocial stressors compound CVD risk, underscoring the complexity of acculturation’s health impact. Cultural and socioeconomic factors – language barriers, healthcare access and intermarriage – emerged as critical determinants. The review calls for disaggregated subgroup analyses and culturally tailored interventions to mitigate CVD disparities among Asian Americans.
Dubois & Giroux, 2025^(21)^	Systematic integrative literature review. No meta-analysis	Socio-economic stressors and psychological mediators. Cultural factors.	This review explores the complex, two-way relationship between nutrition and mental health, specifically focusing on its impact on immigrants in Canada. The authors highlight that diet composition and nutritional status can significantly influence mental well-being (e.g. certain nutrients and dietary patterns are linked to mood and cognitive function), while conversely, mental health conditions like depression or anxiety can affect appetite, food choices and dietary intake. Immigrants are presented as a particularly vulnerable population due to the unique challenges they face. These include migration-related stressors (such as pre-migration trauma, acculturation difficulties, discrimination, socio-economic hardship and loss of social support), which can negatively impact mental health. Simultaneously, immigrants often undergo dietary transitions post-migration, potentially shifting towards less healthy eating patterns due to factors like food availability, cost and time constraints, which can compromise nutritional status. The review emphasises that this bidirectional link can create a detrimental cycle where poor mental health negatively affects nutrition, and poor nutrition negatively affects mental health, with migration stressors acting as key exacerbating factors.
Kim *et al.*, 2025^(45)^	Systematic integrative literature review. No meta-analysis	Socio-economic stressors and psychological mediators. Cultural factors.	This systematic review synthesised 19 US-based studies on neighbourhood racial/ethnic segregation and obesity. Findings reveal consistent positive associations between segregation and obesity among Black and Hispanic adults, particularly Black women, highlighting structural racism and gendered stress as potential mechanisms. Hispanic segregation also predicted higher obesity risk, though effects varied by gender and nativity; foreign-born Hispanics sometimes showed protective effects in low-poverty areas. Neighbourhood poverty emerged as a significant moderator, amplifying segregation’s impact on obesity among US-born Hispanics in high-poverty areas. Mediators such as food environments and perceived access to healthy foods partially explained segregation – BMI links in one study, underscoring the role of built environments. Variability in segregation measures (isolation *v*. dissimilarity indices) and reliance on cross-sectional designs limit causal inference. Future research should employ longitudinal designs to clarify pathways, explore psychosocial mechanisms (stress, belonging) and examine diverse racial/ethnic groups beyond Black and Hispanic populations. Policy implications include addressing structural racism through housing equity, improving food environments in segregated neighbourhoods and promoting residential integration to reduce obesity disparities.
Kronstad *et al.*, 2025^(27)^	Systematic Literature review. No meta-analysis	Physical climate and metabolic/physiological changes. Socio-economic stressors and psychological mediators. Cultural factors.	This scoping review of 20 studies (2013–2023) on overweight/obesity among adult immigrants across the Nordic countries, finding consistently show higher burdens among immigrants than in domestic populations – especially among Somali and Middle Eastern women – and notable heterogeneity by origin, gender and residence duration. Quantitative studies dominate (16/20) with one RCT; four qualitative studies illuminate lived barriers. Cultural factors are central: hospitality norms, traditional high‑calorie foods and gendered restrictions on exercise intersect with acculturation and language proficiency to shape risk. Socio-economic stressors – low education and income, time scarcity, neighbourhood deprivation and limited access to culturally adapted services – constrain diet and activity, while psychological mediators such as winter‑related low mood and emotional eating are reported. The Nordic climate (cold, dark winters) further depresses physical activity, compounding weight gain. Physiologically, immigrant groups show high central adiposity and adverse metabolic profiles; importantly, insulin‑sensitivity‑equivalent cut‑points for BMI and waist circumference appear lower in Iraqi immigrants than in Swedes, implying under‑recognition of risk. Intervention evidence is sparse but promising: culturally tailored, gender‑specific, language‑concordant programs in Sweden achieved modest weight loss and improved insulin sensitivity over four months. The review calls for broader, equity‑oriented strategies integrating language, health literacy, culturally responsive counselling and safe, accessible environments for physical activity across Nordic contexts.
Mendez 2025^(52)^	Systematic literature review and meta-analysis	Physical climate and metabolic/physiological changes. Socio-economic stressors and psychological mediators. Cultural factors.	This meta-analysis synthesises evidence on CVD risk factors among Latino migrant seasonal farmworkers in the USA, a population facing severe health disparities. Across 17 studies, pooled prevalence estimates reveal high burdens of modifiable risks: overweight/obesity (≈57 % for both sexes), hypertension (15 % females; 22 % males), diabetes (13 % females; 12 % males) and high cholesterol (8 % females; 17 % males). Gender differences emerged – males exhibited greater hypertension and diabetes, while females had higher cholesterol. Socio-economic stressors, including poverty, lack of insurance and limited healthcare access, intersect with cultural and occupational factors such as acculturation challenges, gendered work roles and language barriers, shaping health behaviors and outcomes. Physiological vulnerabilities are compounded by harsh physical environments – heat exposure, dehydration and strenuous labour – while psychosocial stress and discrimination further exacerbate risk. The review underscores structural inequities and calls for culturally tailored interventions integrating health education, preventive screening and policy advocacy. Findings highlight the urgent need for equitable cardiovascular health strategies addressing social determinants, occupational hazards and cultural contexts to mitigate chronic disease burdens among Latino migrant workers.
Varre *et al.*, 2025^(49)^	Systematic literature review. No meta-analysis	Physical climate and metabolic/physiological changes. Socio-economic stressors and psychological mediators. Cultural factors.	This review synthesises evidence on dietary acculturation among migrant populations and its health implications. Across 30 studies, migration consistently triggers a shift from traditional diets – rich in whole grains, legumes and vegetables – to host-country patterns dominated by processed foods, added sugars and saturated fats. These changes are associated with increased obesity (5–10 %), type 2 diabetes (7–12 %) and CVD risk. Socio-economic stressors such as low income, food insecurity and limited access to healthy options intersect with cultural and psychosocial factors, including acculturation pressures, loss of traditional food practices and mental health challenges. Physical environments and food systems further shape these transitions, with migrants in high-income countries facing aggressive marketing and limited availability of affordable fresh produce. Physiologically, dietary shifts disrupt gut microbiota diversity, potentially influencing immunity and metabolic health. Cultural identity and social networks modulate these patterns, with ethnic enclaves offering some protection against unhealthy acculturation. The review calls for culturally sensitive interventions—nutrition education, community-based programmes and policy measures like subsidies and food labelling—to preserve healthy traditional diets while supporting adaptation to new environments.
Zheng *et al.*, 2025^(50)^	Systematic literature review. No meta-analysis	Socio-economic stressors and psychological mediators. Cultural factors.	This scoping review synthesises 31 studies on dietary acculturation among Sub-Saharan African immigrants and refugees in the USA. Findings reveal that, despite exposure to Western food environments, many maintain culturally rooted dietary practices – such as consuming fresh produce, traditional grains and home-cooked meals – while adopting some American patterns such as increased meat, fast food and sugar-sweetened beverages. Alcohol intake remains low overall, though Nigerian men report higher consumption than peers in Africa. Food insecurity affects up to 85 % of refugees, driven by financial strain and language barriers, influencing reliance on low-cost starchy staples. Physiologically, obesity prevalence ranges from 27 % to 37 %, with cardiometabolic risk linked to age at immigration and socio-economic stressors. Cultural identity strongly shapes food choices, with adults striving to preserve traditional diets while youth exhibit faster acculturation, favouring processed foods and snacks. Religious norms and social networks buffer unhealthy changes, while intergenerational tensions emerge around meal practices. The review underscores the need for culturally tailored interventions addressing structural barriers, food security and health literacy. Despite qualitative richness, limited quantitative data and inconsistent measures highlight urgent gaps for longitudinal, mixed-method research to inform equitable nutrition policies.

**Table 2.** A table summarising systematically the thirty-three review articles included in the synthesis of this umbrella literature review Table 2 long description.

Review article	Number of databases	Search timeframe	Region of focus	Methods of the represented studies	Instruments or tools used in the reviewed studies	Effect sizes of the included studies
Fernando *et al.*, 2015^(31)^	Two databases: MEDLINE and PubMed	From inception to December 2014	South Asians who immigrated to high-income countries, with studies mainly from Canada, the United Kingdom, the United States, Australia and the Netherlands	Observational studies, randomised clinical trials, non-systematic reviews, systematic reviews and meta-analyses	Administrative and registry data; Mortality databases; Clinical scoring systems; Anthropometric measures (e.g. BMI, waist circumference, waist:hip ratio); Laboratory biomarkers (e.g. ApoB/A1 ratio, HDL/LDL-cholesterol, TAG, HbA1c)	Coronary Artery Disease: prevalence: South Asian migrants have 1·5–2 times higher prevalence than Europids. Diabetes prevalence: South Asians *v*. Europids 1·4–2·5 times higher.
Gasevic *et al.*, 2015^(59)^	Academic Search Premier, Biomedical Reference Collection: Comprehensive, CINAHL, Global Health, MedLine and Social Sciences.	From 2000 to 2014	Immigrants to North America (Canada and United States)	Cross-sectional and longitudinal studies.	National health surveys; Anthropometric measures: BMI, waist circumference; Laboratory measures: cholesterol, blood pressure, TAG, glucose; Self-reported measures: smoking, physical activity, diet.	Hypertension prevalence: Higher in Black individuals *v*. White in almost all studies; Higher in Indigenous people *v*. White in some studies. Lower in Chinese *v*. White in some studies. Diabetes prevalence: Higher in Indigenous, Black, Hispanic, Filipino *v*. White Similar or lower in Chinese *v*. White in some studies. Obesity prevalence: Higher in Indigenous, Black, Hispanic *v*. White Lower in Chinese and Filipino *v*. White
Goulão *et al.*, 2015^(48)^	Three databases searched: PubMed, JSTOR, and EBSCO	From inception until August 2014	Immigrants to United States (25 studies), Canada (4 studies), Netherlands, Spain, Italy, Israel, France, Norway, Luxembourg, Sweden, United Kingdom	Mostly cross-sectional studies, except for one longitudinal study	BMI and overweight prevalence (self-reported or measured) Length of residence in host country (proxy for acculturation) Comparisons with home-country residents Covariates included age, gender, socioeconomic status, language fluency	Hispanic immigrants: 79 % of studies show positive association between length of residence and BMI/overweight. European immigrants: 8 of 11 mentions show positive association (72·7 %). African immigrants: 5 of 9 studies show positive association (55·6 %). Asian immigrants: Only 38·2 % of mentions show positive association; results mixed. Comparative studies: immigrants often have higher BMI than home-country counterparts (differences up to 4–5 kg in some studies).
Kershaw & Albrecht, 2015^(44)^	Three databases searched: MEDLINE, PsycINFO and Web of Science	January 2011–July 2014	Immigrants to the USA	Designs included: cross‑sectional and ecologic studies and one prospective cohort	Segregation measures: Isolation index (exposure dimension at metropolitan level). Percent racial/ethnic composition at census tract/block group/ZIP code. Health measures: Self‑reported behaviours (smoking, diet and physical activity); measured BMI/blood pressure in selected national surveys.	Black segregation: higher metropolitan‑level segregation → higher BMI/obesity, with stronger effects in women; Neighbourhood‑level results mixed, often depending on cut‑points and locales. Hypertension: higher metropolitan‑level segregation associated with greater hypertension prevalence among Blacks. Hispanic segregation: Obesity: mixed; metropolitan isolation associated with lower obesity among Mexican‑American women but higher obesity among Hispanic White women.
Gong & Zhao, 2016^(46)^	PubMed and China National Knowledge Infrastructure (CNKI)	1966 to August 2014	Chinese immigrants worldwide mainly in: United States, Canada, United Kingdom, Mauritius and Internal migrants within China.	Cross-sectional surveys, mortality database analyses and one longitudinal study.	Clinical measures: Stroke and CHD prevalence as well as diabetes, hypertension, dyslipidaemia, obesity and smoking prevalence with data sources including National health surveys (Canada, UK and United States), mortality registries and WHO.	Stroke: Mainland China prevalence: ∼1·9 % *v*. Chinese immigrants: 0·3–0·9 % (Canada, UK). CHD: Mainland China prevalence: ∼0·77 % *v*. Chinese immigrants: 1·7–3·2 % (Canada, UK); Diabetes: Chinese immigrants: 12·7–13·3 % (US) *v*. mainland China: 5·5 %. Canada/UK: lower reported prevalence (∼4 %), likely underestimation due to self-report. Obesity: Chinese immigrants have lower BMI than Whites but higher than mainland Chinese; obesity risk rises with duration of residence.
Wang *et al.*, 2016^(38)^	One database, PubMed	January 1985–April 2015	Refugees immigrating to the USA from Southeast Asia (e.g. Burma, Cambodia, Hmong, Vietnam), Africa (e.g. Sudan, Somalia, Liberia), Middle East and mixed groups.	Cross-sectional and cohort studies were reviewed	Dietary assessment tools: FFQ. 24-h recalls and food diaries. Surveys on food insecurity and acculturation. Acculturation measures: length of stay, language proficiency, cultural orientation scores.	General trends after resettlement in the USA: Increased consumption of meat, eggs, high-fat foods, sweets, fast food and sugar-sweetened beverages. Mixed changes in fruits, vegetables and dairy intake – some groups increased, others decreased. Examples: Cambodian refugees: higher past food deprivation predicted eating fatty meats (OR: 1·14) and overweight/obesity (OR: 1·2). Food insecurity: rates ranged from 24 % (Cambodian) to 85 % (Liberian); associated with low income, low education, low acculturation and language barriers. Acculturation effects: longer US stay linked to more added sugars, oils and processed foods; adolescents influenced by American peers and fast-food culture.
Murphy *et al.*, 2017^(10)^	Two databases: MEDLINE and Google Scholar	2007–2017	International migrants from low- and middle-income countries (LMIC) to high-income countries (HIC), with examples from the USA, UK, Europe and Australia	Systematic reviews and observational studies including cross-sectional, cohort and intervention studies on obesity	Obesity measures: BMI, waist circumference, waist:hip ratio and metabolic syndrome criteria. Acculturation measures: generational status, language use, cultural orientation scales.	Healthy migrant effect: Newly arrived migrants often have lower obesity prevalence than natives. Example: United States-born men had 4 × higher odds of obesity than migrant men and women had 3·5 × higher odds. Weight gain over time: Obesity risk rises sharply after 10–15 years post-migration, often matching or exceeding domestic populations.
Alidu & Grunfeld, 2018^(47)^	Three databases searched: Medline, PsychINFO and EMBASE	January 2001–December 2016	Migrants from low- and middle-income countries (Latin America, Asia, Africa, Middle East) to high-income countries (USA, Canada, UK, Australia, Norway, Netherlands, Sweden and UAE).	Cross-sectional and longitudinal	Acculturation measures: Bidimensional Acculturation Scale for Hispanics, Short Acculturation Scale for Hispanics, Suinn-Lew Asian Self-Identity Scale, Vancouver Index, researcher-developed scales. Proxy indicators: length of stay, language use, generational status. Health behaviour measures: dietary intake (e.g. fast food, fruit/vegetable consumption), physical activity, weight-loss attempts. Obesity measures: BMI (self-reported or measured), waist circumference.	Acculturation and obesity: higher acculturation associated with higher BMI/obesity. Duration of residence: Longer stay linked to higher obesity risk in 17 studies (e.g. OR 4·3 for > 15 years *v*. < 5 years). Generational status: Second-generation migrants often had higher obesity rates than first-generation (e.g. OR 1·76 for Indians; OR 3·65 for Chinese in England). Health behaviours: Greater acculturation associated with unhealthy dietary changes (fast food, sugary drinks and red meat) and lower fruit/vegetable intake. Physical inactivity linked to higher BMI; some studies found protective effects of leisure-time activity among women only. Language: Better host-country language skills correlated with lower obesity risk in several studies.
Ngongalah *et al.*, 2018^(35)^	Nine databases searched: Medline, Embase, PsycInfo, PubMed, CINAHL, Scopus, ProQuest, Web of Science and Cochrane Library	January 1990–February 2018	Immigrants from Morocco, Somalia, Ethiopia, Eritrea, Nigeria, Ghana, Mali, Senegal to high-income countries (Europe, Australia, Canada, Israel).	Reviewed studies included 10 quantitative (9 cross-sectional, 1 longitudinal) and 4 qualitative (focus groups, interviews).	Dietary assessment: FFQ, 24-h recalls, interviews, researcher-developed questionnaires. Physical activity assessment: Self-reported frequency and duration of physical activity (e.g. moderate/vigorous exercise, walking).	Dietary patterns: Bicultural – traditional African foods retained but Western foods adopted (processed meats, snacks and sugary drinks). Three adherence patterns: Strict, flexible, limited traditional diet. Physical activity behaviours: Some studies reported low physical activity levels (65–72 % did not exercise regularly), others reported high physical (87 % exercised ≥ 3 times/week). Barriers to physical activity included cold climate, lack of sidewalks, cultural norms (e.g. modesty, gender segregation), cost of gyms, busy schedules.
Mansour *et al.*, 2020^(37)^	Four databases searched: MEDLINE (Ovid), Embase (Ovid), CINAHL (EBSCO) and PubMed.	From inception to September 2020	Middle Eastern and North African (MENA) immigrants and refugees (e.g. Sudanese and Somali groups) to high income counties, mainly the United States	Only three quantitative cross-sectional studies were reviewed	Food insecurity measurement tools included a modified 10-item Radimer-Cornell Hunger Scale in two reviewed studies and the two items scale from USDA Household Food Security Survey Module in one of the reviewed studies. Additional measures: BMI, body composition, bone density, metabolic biomarkers and physical activity using pedometer.	Prevalence of food insecurity: 67 % among Somali women and 71 % Sudanese families. Determinants included: Recent arrival (< 3 years), low English proficiency, limited education, cultural preference for traditional foods, socio-economic disadvantage. Health impacts for Somali women were that food-insecure participants were 2·66 times more likely to be overweight/obese (OR: 2·66; CI: 1·25, 5·69; *P* = 0·01). A paradox was observed whereby food insecurity is linked to both undernutrition (wasting, low bone mass – especially among children) and overweight/obesity.
Amiri, 2021^(2)^	Two databases searched: PubMed and Embase.	Inception to February 2020	Immigrant and refugee populations across continents (Europe, America, Asia and Australia).	Included 59 cross-sectional and longitudinal quantitative studies on immigrants and refugees aged ≥ 15 years.	BMI-based classification: Overweight: BMI ≥ 25 Obesity: BMI ≥ 30 Data extracted from measured and self-reported BMI.	Overall prevalence: BMI ≥ 25 (overweight + obesity): 49 % (95 % CI: 42, 56 %). Obesity: 24 % (95 % CI: 22, 27 %). Overweight: 37 % (95 % CI: 31, 44 %). By gender: Men: Obesity 20 %, Overweight 44 %. Women: Obesity 27 %, Overweight 34 %. By migration status: Immigrants: Obesity 24 %, Overweight 38 %. Refugees: Obesity 23 %, Overweight 29 %. By measurement method: Measured obesity: 25 % *v*. self-reported 23 %. By continent: Obesity: America 28 %, Europe 21 %, Asia 23 %, Australia 20 %. Overweight: Europe 38 %, America 38 %, Asia 33 %, Australia 40 %.
Bains *et al.*, 2021^(33)^	Seven databases searched: MEDLINE, Embase, Cochrane Library, CINAHL, PsycINFO, Maternity & Infant Care Database and SveMed+	Between 2000 and 2019	Immigrant women’s prenatal health in Norway	Included 44 peer-reviewed articles with 8 qualitative studies and the remainder quantitative.	Common measures in included studies: BMI, gestational diabetes screening, blood pressure, vitamin D and Fe biomarkers, dietary intake and qualitative interviews on health service experiences.	Women from Africa/Middle East had higher gestational weight gain (up to +2·7 kg); South Asian women who are less physically active had more abdominal obesity. Severe vitamin D deficiency: 45 % (South Asia), 40 % (Middle East), 26 % (sub-Saharan Africa) *v*. 1·3 % (Western Europe).
Ankomah *et al.*, 2022^(60)^	Eight databases searched: Ovid MEDLINE, EMBASE, PsycINFO, CINAHL, ProQuest, Scopus, PubMed and Web of Science. Grey literature and reference lists were also reviewed.	From inception until February 2022	Immigrants and refugees originating from low- and middle-income countries (LMIC)in high-income countries (HIC) including United States, Switzerland, Greece and Australia.	Included 5 observational studies (4 cross-sectional, 1 mixed-methods).	Anthropometric measures of weight based on WHO standards (height-for-age, weight-for-age, weight-for-height and BMI-for-age). Data collection included medical record reviews, face-to-face interviews.	Double burden of malnutrition (DBM): coexistence of undernutrition and overnutrition in the same population. Overnutrition prevalence (overweight/obesity): Range: 11·1–42 % across studies. Example: Geneva refugees – overweight 42 %. Undernutrition prevalence (stunting, wasting and underweight): Range: 0·3–17 %. Example: Syrian child refugees—stunting 17 %, wasting 1·9 %. Micronutrient deficiencies include Vitamin D deficiency and anemia, which was common in refugee children in the United States.
Berggreen-Clausen *et al.*, 2022^(36)^	Three databases were searched: EMBASE, PubMed and Web of Science.	From 2007 to May 14, 2021	High‑income countries (HIC); 68 included studies were primarily from the USA (*n* 45), with others from Canada (*n* 10), Australia (*n* 7), the UK (*n* 3), Switzerland (*n* 1), Norway (*n* 1) and the Netherlands (*n* 1). Populations originated from Asia, Africa, the Middle East, South/Central America and the Caribbean.	68 studies synthesised: 42 qualitative, 9 quantitative, 17 mixed‑methods; ∼35 % women‑only samples.	Statistical analysis and prevalence calculations were used in quantitative studies and interviews, focus groups and ethnographic methods were used in qualitative studies.	Physical environment: Immigrants in low‑income areas reported high availability of fast food and energy‑dense options, lower quality/variety of produce in nearby stores and reliance on multiple outlets (supermarkets + ethnic shops; farmers’ markets; flea markets; foraging/home gardens) to obtain acceptable, affordable foods. Economic environment: Low income and competing necessities (rent, utilities and remittances) constrained food choice; healthy/cultural and halal foods were costlier; larger, distant stores were cheaper but required transport. Socio‑cultural environment: Strong values for fresh, chemical‑free, unprocessed, traditional foods; scepticism towards packaged/frozen foods; religious rules (e.g. halal) shaped availability perceptions. Time scarcity (double work burden, long hours/commutes) → convenience foods, eating out, skipped meals. Mobility (car access, public transport limits, weather, carrying capacity) → ability to reach cheaper/better outlets. Navigation (language/label literacy, unfamiliar market formats, social‑network guidance) → progressive improvement over time; early periods especially challenging.
Chapagai & Fink 2022^(61)^	Five databases were searched: MEDLINE (via PubMed), CINAHL, Embase, PsycINFO and Scopus; grey literature was also searched.	From inception to July 2022	Immigrants from South Asia, India, Pakistan, Bangladesh and Nepal to high-income countries including UK, United States, Canada and Netherlands	All 23 reviewed studies used quantitative methods such as correlation/associations analysis, case–control studies, descriptive exploratory, longitudinal and secondary data analyses	Cardiovascular-related measures included blood pressure and clinical medical history, Laboratory tests (e.g. Hb, lipid profiles).	Short sleep and coronary events (India): Sleeping < 6 h/night associated with ∼threefold increased risk of coronary events. Sleep duration/symptoms and chronic disease (Bangladesh national survey): Short sleep associated with chronic disease: RR = 1·1 (95 % CI 1·05, 1·28).
Ismail *et al.*, 2022^(32)^	Six databases were searched: Scopus, MEDLINE (Ovid), CINAHL, Embase, ASSIA, Cochrane Library; grey literature was searched via Web of Science	From inception until February 2021	Immigrants living in the United Kingdom	Studies reviewed included observational (cross‑sectional, case‑control, cohort) and experimental; the 10 included studies comprised cross‑sectional (*n* 5), cohort/longitudinal (*n* 2) and secondary analyses of national datasets (*n* 3)	Health outcomes included obesity/anthropometry (BMI, waist circumference, abdominal obesity; child overweight trajectories).	Example: Ghanaian migrants in London *v*. rural Ghana: Obesity prevalence ratio (men) PR = 15·04 (95 % CI 5·98, 37·84); women PR = 6·63 (95 % CI 5·04, 8·72). Abdominal obesity (men) PR = 10·48 (95 % CI 4·43, 24·77); women PR = 2·56 (95 % CI 2·25, 2·91).
Matsangos *et al.*, 2022^(34)^	Medline, PubMed, ScienceDirect, UNHCR, Eurostat, OCHA, International Organization of Migration (IOM), BMC Public Health, PLoS Med, OECD, Eurofound, PLoS One and WHO	From 1994 to May 2022	Afghan refugees settled in Europe (Germany, Austria, Hungary, Sweden, Switzerland highlighted as top destinations).	Integrative review including academic articles, literature reviews and grey literature; any design (observational, cross-sectional, retrospective) considered.	Health indicators: prevalence of infectious diseases (hepatitis, malaria, leishmaniasis and intestinal parasites), non-communicable diseases (hypertension, diabetes, obesity), mental health and women’s health access. Risk factors: malnutrition, vitamin D deficiency, smoking, low socio-economic status, poor hygiene, trauma exposure.	Obesity prevalence: 31·2 % among Afghan adults; overweight 32·1–38 %. Hypertension: 46·2 %; diabetes: 13·3 %; lipid abnormalities: ∼70 %.
Mensah *et al.*, 2022^(42)^	Six databases were searched: PubMed, Embase.com, CINAHL Plus, Cochrane Library (via Wiley), Scopus and Web of Science	From inception to 19th July 2021	Immigrants from sub‑Saharan African (SSA) countries residing in high‑income countries including Europe, United States, Australia, New Zealand and Israel.	Observational cross‑sectional and cohort studies were reviewed	The reviewed studies assessed hypertension, diabetes, obesity, smoking, dyslipidaemia and CVD.	Hypertension: 26 % overall (range 6–55 %); Diabetes: 11 % overall (examples: 4·5 % among Somalians in the USA to 17·4 % among Ethiopians in Israel. Overweight/Obesity: 59 % overall; where separated, average overweight 34 % and obesity 30 %; generally higher in women than men.
Osei *et al.*, 2022^(43)^	Two databases: PubMed and LIVIVO	From inception to November 2020	Immigrants living in Germany	Quantitative studies including cross-sectional, cohort, case–control and ecological studies	Immigrants identified from registry data and clinical measures used for BMI and other measures.	No pooled effect sizes were performed due to heterogeneous exposures. Associations were synthesised as positive/negative/null (heat map), not as pooled estimates.
LeCroy *et al.*, 2023^(41)^	One database: PubMed	From 2000 to March 2022	Immigrants to the United States	Included quantitative, qualitative and mixed‑methods studies on cardiometabolic disease (CMD), diet and immigration.	The reviewed articles utilised surveys/datasets on hypertension, overweight/obesity and diabetes by region/country of birth). Qualitative tools included semi‑structured interviews and focus groups.	No pooled estimates were produced; this review is narrative, integrating prevalence and comparative findings from many sources alongside qualitative themes.
Lopez *et al.*, 2023^(51)^	Two databases: PubMed and SCOPUS.	From inception to May 2021	Immigrants from lower middle-income countries (LMIC) such as African, Asian, South American and Mexican to high-income countries (HIC) such as Western Europe and North America.	Included original observational quantitative studies (cohort and cross‑sectional).	Statistical analysis to estimate prevalence.	Pooled effect sizes: Type 2 diabetes (T2D), migrants *v*. host: PR 1·48 (95 % CI 1·35, 1·65). T2D, migrants *v*. origin: PR 1·80 (95 % CI 1·40, 2·34); strongest for African migrants. Obesity, migrants *v*. host: PR 0·89 (95 % CI 0·80, 1·01) (overall not higher; Asians lower). Obesity, migrants *v*. origin: logPR 0·75 (≈PR 2·12) overall; reduced to logPR 0·12 (≈PR 1·13) when very large effects excluded. Hypertension, migrants *v*. host: Centered on null (logPR 0·07; 95 % CI –0·10, 0·15) with subgroup variations (e.g. African migrants higher).
Paixão *et al.*, 2023^(28)^	Six databases searched: PubMed, Web of Science, Embase, Lilacs, Scielo and Google Scholar	Studies published between 1980 and June 2020	Latino immigrants residing in the USA (including Mexicans, Central and South Americans; very few studies on Brazilians).	Quantitative studies: 55 cross-sectional, 4 cohort and 1 case–control.	Anthropometric measurements included height and weight measured using calibrated stadiometers and scales to calculate BMI; waist circumference for abdominal obesity, following WHO protocols. Self-reported measures using surveys and administrative/contextual data were also employed in the reviewed articles.	Pooled estimates: Hypertension: 28 % (95 % CI: 23, 33 %). Type 2 Diabetes: 17 % (95 % CI: 14, 20 %). General obesity (BMI ≥ 30): 37 % (95 % CI: 33, 40 %). Abdominal obesity: 54 % (95 % CI: 48, 59 %). Metabolic syndrome: 39 % (95 % CI: 32, 45 %). Low HDL-cholesterol: 42 % (95 % CI: 35, 49 %). High TAG: 38 % (95 % CI: 24, 51 %).
Ufholz & Werner 2023^(40)^	One database: PubMed	Publications between 2018 and 2023	Primarily the United States with inclusion of international studies (e.g. Canada, Russia, Saudi Arabia, Greece); Not focusing on immigrants only, but included comparison between immigrants and domestic populations.	Review included original empirical studies (mostly cross‑sectional, plus longitudinal cohort) that examined fast‑food (FF) intake and its social/demographic correlates.	Dietary assessment: 24‑h recalls with food source identification; Single‑ or few‑item frequency questions (e.g. ‘How many times did you eat fast food in the past week?’), often naming brands/food types, Multi‑item checklists of FF categories (burgers, pizza, fried chicken, etc.); Clinical measures in several cohorts: BMI, waist circumference, fat depots (visceral, hepatic fat) and metabolic biomarkers.	Non‑Hispanic Black adults more likely to consume FF; mean kcal/day from FF highest among younger adults; example adult mean ∼309 kcal/d; ages 20–39 ∼425 kcal/d, 40–59 ∼323 kcal/d, ≥ 60∼157 kcal/d. Immigrant Latinos (especially undocumented women) report lower FF frequency than U.S.‑born Latinos.
Kibibi *et al.*, 2024^(30)^	Three sources: PubMed, Embase and OpenGrey.	From inception to September 2021	Refugees settling in High-income countries including United States, Australia, South Korea, New Zealand, Turkey, Sweden	9 observational studies (6 cross-sectional, 2 retrospective cohorts, 1 prospective cohort).	Refugee status determined via: Medical records linked to public health databases; Social service program records; Immigration/census data; and Self-reported in surveys. Outcome measurement (obesity): Anthropometric measures: BMI ≥ 25 kg/m^2^ for adults; WHO/CDC growth standards for children using medical records or self-reported height/weight.	Main pooled effect: OR = 1·11 (95 % CI: 0·56, 2·20), I^2^ = 97 % → No significant overall association between refugee status and obesity. Subgroup analyses: Adults only: OR = 1·59 (95 % CI: 0·82, 3·06), I^2^ = 97 %. Men only: OR = 0·72 (95 % CI: 0·57, 0·89), I^2^ = 45 % → Significant lower odds among refugee men. Women only: OR = 1·72 (95 % CI: 0·92, 3·24), I^2^ = 95 %. U.S. studies: OR = 0·52 (95 % CI: 0·08, 3·54), I^2^ = 99 %. Non-United States: OR = 1·63 (95 % CI: 0·72, 3·70), I^2^ = 94 %. Asian *v*. non-Asian high-income countries: OR ≈ 1·02 *v*. 1·16 (both non-significant).
Nayak *et al.*, 2024^(62)^	Seven databases: PubMed, CINAHL, Web of Science, PsycINFO, Academic/Nursing and Social Work Abstracts and Google scholar	Between 2010 and 2024	Undocumented immigrants from Asian countries residing in the USA	13 empirical studies (9 quantitative, 4 qualitative). All were cross-sectional or pooled secondary data analyses; no longitudinal studies identified.	Quantitative studies: Self-rated health measures (e.g. Likert scales). Standardised survey instruments for mental health (depression scales), health insurance, chronic conditions and obesity. Secondary data from NHIS and SIPP for chronic disease and BMI. Qualitative studies: Semi-structured interviews and focus groups exploring psychosocial needs, healthcare access and perceptions.	No pooled effect sizes; Narratively summarised mental health; Self-rated health differences between undocumented Asians and Latinos for example, Chronic conditions prevalence (diabetes, hypertension, heart disease, asthma and cancer) and obesity in national datasets.
Purcino & Bedrikow, 2024^(39)^	Six databases: LILACS, MEDLINE, Scopus, Web of Science, Cochrane, Embase, plus thesis/dissertation repositories	From inception until July 2023	Haiti immigrants mostly in the United States and Canada.	Review included primary research (cross-sectional, ethnography, qualitative interviews, focus groups).	Dietary quality indexes (Healthy Eating Index, Alternate Healthy Eating Index). FFQ, 24-h recalls. Ethnographic observation and qualitative interviews for cultural food practices. Acculturation measures included length of stay, language proficiency, self-reported dietary changes. Health measures included anthropometry (BMI, waist circumference), indicators of metabolic syndrome and obesity prevalence.	Obesity prevalence: Adults: 43 %; women: 50 % (higher than Latinas 39 %, Brazilians 25 %). Metabolic syndrome: 8–13 % of subjects. Associations: Food hardship linked to higher obesity odds. Dietary quality positively associated with > 15 years in United States and English proficiency.
Vo *et al.*, 2024^(29)^	Two databases: PubMed and Google Scholar.	Between 1990 and 2022	Immigrants in United States, specifically Asian American immigrants (focus on Southeast Asians but also included South and East Asians due to limited data).	Observational studies including cross-sectional and cohort	Acculturation proxies: English spoken at home. Length of stay in the United States. Religiosity/spirituality (frequency of prayer, religious participation). Admixed family structures (interracial marriage). Health measures via Self-reported surveys. Anthropometric measures: BMI, waist:hip ratio, HbA1C, blood pressure and lipid profiles. Behavioural measures included smoking, diet, physical activity and caloric intake.	Length of stay & diabetes: ≥ 15 years in United States → OR for diabetes = 1·5 (95 % CI: 1·3, 1·7); OR for prediabetes = 1·3 (95 % CI: 1·2, 1·6). Religiosity (MESA): Higher religious practice linked to higher systolic BP (127·5 mmHg daily *v*. 121·5 mmHg never) and higher BMI (29·3 *v*. 26·4 kg/m^2^). Acculturation strategies (MASALA): Women in assimilation group had lowest obesity prevalence (26·5 %) and diabetes (16·4 %). Integration group had lowest prediabetes (29·7 %). Assimilation linked to higher exercise and lower caloric intake.
Dubois & Giroux, 2025^(21)^	Six databases: MEDLINE, CINAHL, Embase, PsycINFO, Scopus, Web of Science.	From inception to July 2024	Immigrants to Canada	Review included quantitative, qualitative and mixed-methods studies	Dietary measures: Healthy Eating Index, dietary recalls and FFQ. Mental health measures: Depression and anxiety scales, psychological distress questionnaires. Acculturation proxies: Length of stay, language proficiency, cultural food practices. Gut microbiome studies: Fecal microbiota analysis, probiotic interventions.	Obesity & mental health: Obesity increases depression risk by 55 % and depression increases obesity risk by 58 %. In a prospective cohort study: Depression predicted obesity; obesity predicted later depression (OR up to 6·27 for anxiety in obese women). Mediterranean diet is protective; Western diet increases depression risk. In a prospective cohort study: Improved diet quality leads to better mental health. Gut microbiome: Fecal transplant from major depressive disorder patients induced depression-like behaviours in mice; probiotics reduced anxiety and distress in clinical trials. Immigrant-specific findings: food insecurity prevalence among Ottawa immigrant mothers: 45 %; recent immigrants (≤ 5 years) most affected. Decline in diet quality 2–5 years post-settlement results in poorer mental health compared to domestic-born Canadians. Gut microbiome diversity loss post-immigration linked to obesity and increased CVD.
Kim *et al.*, 2025^(45)^	Five databases: PubMed, PsycINFO, Dissertations and Theses Global, CINAHL, Academic Search Complete.	From inception to February 2023	Immigrants in the United States	Review included quantitative cross-sectional and longitudinal studies.	The studies used data from direct measurement, self-reported and medical records to assess obesity, BMI and waist circumference.	Eight of nine studies found positive association between Black segregation and obesity risk, especially among Black women. Four studies reported significant positive association between Hispanic segregation and obesity risk. Neighbourhood poverty was significant for US-born Hispanics (segregation effect stronger in high poverty areas). Neighbourhood food environment and perceived access to healthy foods mediated segregation – BMI link in one study.
Kronstad *et al.*, 2025^(27)^	Four databases: CINAHL, PsycINFO, PubMed, Web of Science	From 1 January 2013 to 31 December 2023	Immigrants residing in Nordic countries (Sweden, Norway, Finland, Denmark, Iceland).	16 quantitative studies (cross‑sectional and cohort/secondary analyses). 4 qualitative studies (interviews, focus groups, ethnography).	Anthropometry: BMI, waist circumference, waist:hip ratio, waist‑height/hip‑height ratios, abdominal diameter. Clinical/biomarkers: insulin sensitivity/resistance, lipids (LDL/HDL, TAG), blood pressure, plasma, etc… Questionnaires/assessments: self‑reported Norwegian language proficiency as proxy for acculturation; physical activity and diet questionnaires.	Somali women *v*. Finnish women obesity PR 1·68 (95 % CI 1·40, 2·03); Kurdish women PR 1·41 (1·15, 1·72). Poor/medium Norwegian proficiency associated with higher odds of overweight OR 1·20 (95 % CI 1·03, 1·40). Somali women *v*. men overweight/obesity OR 6·13 (95 % CI 2·81, 13·4); residence ≥ 14 years *v*. ≤ 4 years OR 7·16 (95 % CI 2·14, 23·8). Longer residence and poor language proficiency linked to higher obesity. Overweight/obesity among immigrants in Norway 53–65 % depending on sample; Somali women obesity up to 54·1 %; Iraqi women 37·1 %, Iraqi men 33·4 %.
Mendez 2025^(52)^	Multiple sources were used: Google Scholar, Web of Science, published peer-reviewed reports, large datasets	From inception to February 2023	Latino migrant seasonal farmworkers in the United States	10 descriptive, 6 cross-sectional, 1 prevalence study.	Clinical measures: Blood pressure, BMI, cholesterol, diabetes screening. Self-reported surveys: Lifestyle, health behaviours, acculturation factors and electronic health records for large samples.	High cholesterol: Female: Event rate 0·08; 95 % CI 0·04, 0·15; PI 0·01–0·54 Male: Event rate 0·17; 95 % CI 0·12, 0·23; PI 0·05–0·43 Diabetes mellitus: Female: 0·13; CI 0·08, 0·21; PI 0·02–0·57 Male: 0·12; CI 0·07, 0·21; PI 0·01–0·63 Hypertension: Female: 0·15; CI 0·10, 0·22; PI 0·03–0·54 Male: 0·22; CI 0·15, 0·31; PI 0·04–0·68 Overweight/Obesity: Female: 0·57; CI 0·26, 0·84; PI 0·01–1·00 Male: 0·57; CI 0·29, 0·81; PI 0·01–0·99
Varre *et al.*, 2025^(49)^	Three databases: PubMed, Scopus, Web of Science	From January 2000 to April 2024.	Global perspective – migrant populations across multiple continents and cultural contexts	30 studies observational quantitative (cross-sectional and cohort) and qualitative studies	Dietary assessment tools: FFQ, 24-h recalls, acculturation scales. Health measures: BMI, blood pressure, diabetes markers, cardiovascular risk indicators.	Increased processed food intake by 15–20 % among immigrants. Increased obesity by 5–10 % and type 2 diabetes by 7–12 % post-migration. Length of residence, age at migration, socio-economic status and food environment also play a role in the risk of obesity.
Zheng *et al.*, 2025^(50)^	Three databases: PubMed, CINAHL, Scopus	From January 2000 to September 2022	Immigrants and refugees from Sub-Saharan Africa residing in the United States	31 studies included involving 17 qualitative (interviews, focus groups, photovoice), 8 quantitative (surveys, secondary data analysis) and 6 mixed-methods.	Dietary assessment: food diaries (3-day), surveys, interviews, photovoice, national health datasets. Acculturation measures: language proficiency, length of stay, proxy indicators. Anthropometric measures: BMI, waist circumference, blood pressure. Food security tools: Household Food Security Survey Module.	BMI & obesity prevalence: Nigerian immigrants: Obesity 37 %, overweight 28·7 %. Africans arriving before age 20: Mean BMI 27·6 kg/m^2^, obesity 27 %. Food insecurity: Refugees reported 25–85 % prevalence; recent arrivals ≤ 3 years more prone to insecurity. Dietary changes: Increased meat and fast-food intake; decreased traditional grains; soda and sweetened beverage consumption rose with longer stay.

**Figure 1:**
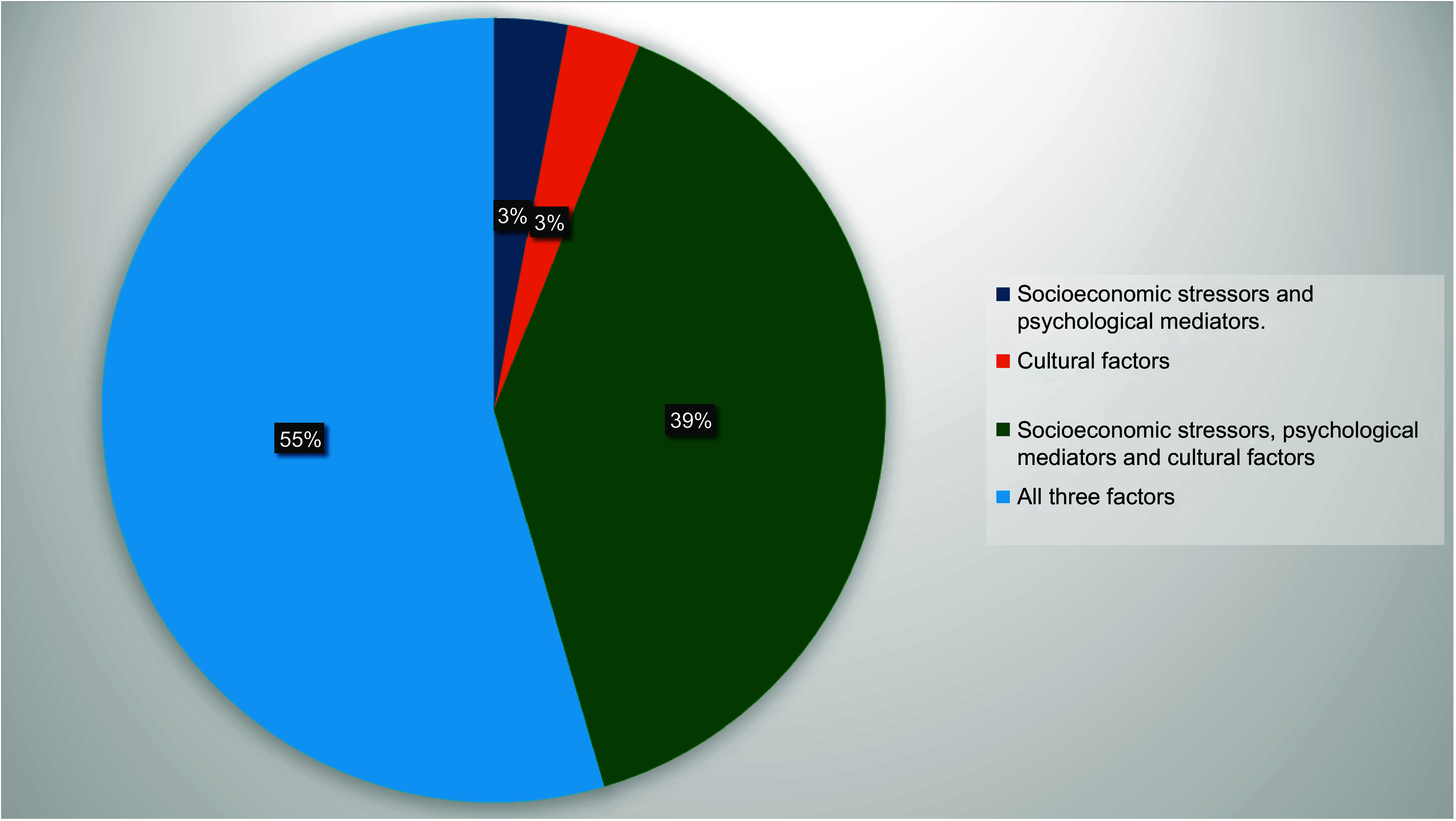
Figure 1 long description. The representation of the three themes in the thirty-three review articles.

Figure 1 summarises how the three themes influencing obesity and weight gain among immigrants in high-income countries are represented across the thirty-three review articles. Most reviews (*n* 18, 55 %) addressed all three themes, thirteen (39 %) examined both socio-economic stressors/psychological mediators and cultural factors, and only one review focused exclusively on either cultural or socio-economic factors.

### Physical climate and metabolic/physiological changes

Eighteen of the thirty-three included reviews examined the role of physical climate and physiological changes in shaping obesity and weight gain among immigrants to high-income countries. Specifically, migration to high-income countries was consistently associated with significant metabolic and physiological changes that predispose immigrants to obesity and related cardiometabolic risks. Evidence from multiple systematic reviews indicates that immigrants often arrive with a health advantage – lower BMI and reduced cardiometabolic risk – yet this advantage diminishes over time, with obesity prevalence rising sharply after 10–15 years post-migration^(10,27)^. Duration of residence emerged as a strong predictor of weight gain, with longer stays linked to higher BMI and increased odds of diabetes and metabolic syndrome^(28,29)^. Furthermore, meta-analyses in some of the studies confirm elevated prevalence of metabolic syndrome (33·1 %) and abdominal obesity (44·8 %) among Latino immigrants in the USA^(28)^, while pooled estimates for refugees suggest heterogeneous obesity risk, with subgroup differences by sex and region^(30)^.

Physiological vulnerabilities discussed in the reviewed articles included greater visceral adiposity and insulin resistance at lower BMI thresholds among South Asian and African migrants compared to host populations^(31,32)^. Reviews also highlighted micronutrient deficiencies – particularly vitamin D and folate – among pregnant women and refugees, compounded by reduced sun exposure and dietary changes in colder climates^(33,34)^. Harsh physical environments, such as cold Nordic winters, were reported to limit outdoor physical activity, further contributing to weight gain^(27)^. Ngongalah *et al.* (2018) by focusing on African women of childbearing age also found that physical activity levels varied widely, with some women reporting reduced engagement due to environmental constraints, cultural beliefs and competing responsibilities such as childcare and employment, which would consequently impact obesity and weight gain prevalence post-immigration^(35)^. Environmental barriers were also noted in Berggreen-Clausen *et al.* (2022), where immigrants reported difficulties accessing fresh, culturally appropriate foods due to poor neighbourhood infrastructure, limited public transport and adverse weather conditions. These challenges were compounded during colder seasons, which restricted mobility and access to healthier food outlets^(36)^. Collectively, these findings emphasise the complex interplay between climate, physiology and environmental constraints in shaping obesity risk among immigrant populations.

### Socio-economic stressors and psychological mediators

Socio-economic and psychological factors emerged as central themes in thirty-two of the reviewed articles. Immigrants to high-income countries often face significant socio-economic disadvantages, including poverty, food insecurity, unstable employment, low income,and limited access to healthcare. These stressors were consistently linked to limited access to healthy foods with increased reliance on energy-dense, processed options and increased obesity risk^(36,37)^. Purcino & Bedrikow (2024) and Wang *et al.* (2016) documented how Haitian and refugee populations, respectively, experienced a shift away from traditional diets rich in fruits, vegetables and legumes toward processed, calorie-dense foods. This transition was driven by economic constraints, time limitations and lack of nutritional knowledge. Food insecurity was a pervasive issue, exacerbated by language barriers, discrimination and difficulties navigating food assistance programs^(38,39)^. Food insecurity prevalence was also high among Middle Eastern and African migrants in the USA reaching 40–71 % and correlating with higher odds of overweight and obesity^(37)^. Employment instability and time scarcity were linked as well to greater fast-food consumption, particularly among working-age adults, thus contributing to a higher prevalence of weight gain^(40)^. LeCroy *et al.* (2023) added to the discussion by emphasising immigration as a structural determinant of health. The review showed that systemic barriers – such as inadequate healthcare access, discriminatory policies and poor neighbourhood food environments – create persistent obstacles to maintaining healthy lifestyles. These structural inequities contribute to cardiometabolic disparities across diverse immigrant groups, reinforcing the need for policy-level interventions^(41)^. Gender disparities were also noted by Mensah *et al.* (2022) among African immigrants in high-income countries, with women exhibiting higher rates of obesity and men showing elevated prevalence of hypertension and diabetes^(42)^.

Additionally, psychological mediators – including acculturative stress, discrimination and migration-related trauma – were frequently cited as indirect drivers of unhealthy behaviours and weight gain^(21,43)^. Mental health challenges such as depression and anxiety were shown to interact bidirectionally with diet quality, creating cycles that exacerbate obesity risk^(21)^. Structural stressors, including residential segregation and neighbourhood deprivation, amplified obesity disparities among Black and Hispanic immigrants in the USA^(44,45)^. These findings underscore the interplay between socio-economic and psychosocial factors in shaping obesity trajectories post-migration. Finally, it is worth to note that longer duration of residence in the host country was frequently reported by several systematic reviews to increase the risk of obesity and obesity-related outcomes, suggesting cumulative exposure to socio-economic and psychological stressors^(27,28,46,47)^.

### Cultural factors

Cultural influences were addressed in thirty-two reviews, with particular emphasis on acculturation, generational differences and dietary transitions. A consistent finding across multiple reviews^(27,28,46–48)^ was the positive association between longer duration of residence and increased BMI and obesity risk. Immigrants often arrive in host countries with lower BMI compared with domestic-born populations (in line with the healthy migrant effect theory), but this health advantage diminishes over time, often within the first decade of residence. This is due to cultural adaptation, which is a critical determinant of dietary and physical activity behaviours over time. Most reviews reported that immigrants retain elements of traditional diets while progressively adopting host-country patterns characterised by higher intake of fats, sugars and processed foods^(49,50)^. Language proficiency and generational status were also noted as acculturation measures that affect BMI and obesity risk^(29,47)^. For example, second-generation migrants often exhibited higher obesity prevalence and more unhealthy behaviours than first-generation counterparts^(47)^. This was mainly attributed to unhealthy behaviours among the second-generation immigrants such as a higher rate of tobacco smoking, alcohol consumption, snack foods and sweet foods overeating, as well as lower levels of physical activity^(47)^. On the other hand, language proficiency was associated with a lower risk of obesity, suggesting that social integration for all immigrant generations may have protective effects in some contexts^(47)^.

The risk of obesity and weight gain was also influenced through cultural norms and gender roles that impact physical activity, with restrictions on women’s mobility and modesty norms limiting exercise opportunities in some groups^(27,35)^. Religious practices and food preferences also shaped dietary choices, sometimes creating tension between cultural identity and health-promoting behaviours^(36)^. Ethnic enclaves provided partial protection against unhealthy acculturation by facilitating access to traditional foods, though these environments were often characterised by high poverty and limited healthcare access^(44)^.

### Methodological quality evaluation of the systematic review articles

The methodological quality of the included reviews was assessed using the A Measurement Tool to Assess Systematic Reviews tool^(24–26)^. Among the six systematic reviews with meta-analysis, three achieved high quality (scores ≥ 8)^(28,30,42)^ and three scored moderate quality (scores 4–7)^(2,51,52)^. These reviews demonstrated rigorous methodology, including comprehensive literature searches, duplicate data extraction and appropriate use of quality assessments and statistical methods. The weaknesses of these systematic reviews involved limited reporting of publication bias and conflict of interest (Table 3).

**Table 3.** A measurement tool to assess systematic reviews (AMSTAR) summary quality assessment for the six systematic review articles that included a meta-analysis Table 3 long description.

	Systematic reviews that included meta-analysis					
AMSTAR question	Amiri, 2021^(2)^	Mensah *et al.*, 2022^(42)^	Lopez *et al.*, 2023^(51)^	Paixão *et al.*, 2023^(28)^	Kibibi *et al.*, 2024^(30)^	Mendez 2025^(52)^
‘A priori’ design provided	*	*	*	*	*	*
Duplicate study selection and data extraction	*	*		*	*	
Comprehensive literature search	*	*	*	*	*	*
Publication status as an inclusion criteria		*	*	*	*	
List of studies (included and excluded) provided			*			
Characteristics of the included studies provided	*	*	*	*	*	*
Quality assessment	*	*		*	*	*
Quality used appropriately	*	*		*	*	*
Methods used to combine appropriate	*	*	*	*	*	*
Publication bias assessed				*	*	
Conflict of interest stated						
Total score	7 (moderate quality)	8 (high quality)	6 (moderate quality)	9 (high quality)	9 (high quality)	6 (moderate quality)

* Indicates that the systematic review article possesses this quality and is awarded one point on the AMSTAR scale.

For the twenty-seven systematic reviews without meta-analysis, quality ranged from low (score ≤ 3) to moderate (scores 4–7). Only one systematic review by Mansour *et al.* (2020) was rated as high quality^(37)^. Common strengths included clear inclusion criteria, comprehensive searches and provision of study characteristics; whereas frequent limitations included lack of duplicate data extraction, incomplete lists of included/excluded studies and insufficient assessment of publication bias and conflict of interest disclosures of the reviewed studies. Only a minority of reviews integrated quality assessments into the interpretation of findings (Table 4). These methodological gaps highlight the need for more rigorous designs, transparent reporting and standardised approaches in future reviews to strengthen evidence synthesis on migration-related obesity and weight gain. Nevertheless, despite the observed methodological quality variation, the collective evidence provides a robust and multifaceted understanding of the cultural, socio-economic, psychological, physiological and environmental determinants of obesity among immigrants to high-income countries.

**Table 4.** A measurement tool to assess systematic reviews (AMSTAR) summary quality assessment for the 27 systematic review articles that did not include a meta-analysis Table 4 long description.

	AMSTAR question											
Systematic review articles	‘A priori’ design provided	Duplicate study selection and data extraction	Comprehensive literature search	Publication status as an inclusion criteria	List of studies (included and excluded) provided	Characteristics of the included studies provided	Quality assessment	Quality used appropriately	Methods used to combine appropriate	Publication bias assessed	Conflict of interest stated	Total score
Fernando *et al.*, 2015^(31)^	*		*						NA			2 (low quality)
Gasevic *et al.*, 2015^(59)^	*	*	*						NA			3 (low quality)
Goulão *et al.*, 2015^(48)^	*	*	*	*		*	*		NA			6 (moderate quality)
Kershaw & Albrecht, 2015^(44)^	*		*			*			NA			3 (low quality)
Gong & Zhao, 2016^(46)^	*		*	*		*			NA			4 (moderate quality)
Wang *et al.*, 2016^(38)^	*	*	*			*			NA			4 (moderate quality)
Murphy *et al.*, 2017^(10)^	*		*	*					NA			3 (low quality)
Alidu & Grunfeld, 2018^(47)^	*	*	*	*		*			NA			5 (moderate quality)
Ngongalah *et al.*, 2018^(35)^	*	*	*	*		*	*	*	NA			7 (moderate quality)
Mansour *et al.*, 2020^(37)^	*	*	*	*		*	*	*	NA	*		8 (high quality)
Bains *et al.*, 2021^(33)^	*	*	*						NA			3 (low quality)
Ankomah *et al.*, 2022^(60)^	*	*	*	*		*	*	*	NA	*		7 (moderate quality)
Berggreen-Clausen *et al.*, 2022^(36)^	*	*	*	*		*			NA			5 (moderate quality)
Chapagai & Fink 2022^(61)^	*		*	*		*	*	*	NA			6 (moderate quality)
Ismail *et al.*, 2022^(32)^	*	*	*	*		*	*	*	NA			7 (moderate quality)
Matsangos *et al.*, 2022^(34)^	*		*	*					NA			3 (low quality)
Osei *et al.*, 2022^(43)^	*	*	*			*	*	*	NA	*		7 (moderate quality)
LeCroy *et al.*, 2023^(41)^	*		*	*					NA			3 (low quality)
Ufholz & Werner 2023^(40)^	*		*			*			NA			3 (low quality)
Nayak *et al.*, 2024^(62)^	*	*	*	*		*			NA			5 (moderate quality)
Purcino & Bedrikow, 2024^(39)^	*	*	*	*		*			NA			5 (moderate quality)
Vo *et al.*, 2024^(29)^	*		*			*			NA			3 (low quality)
Dubois & Giroux, 2025^(21)^	*	*	*	*		*	*	*	NA			7 (moderate quality)
Kim *et al.*, 2025^(45)^	*	*	*	*		*	*	*	NA			7 (moderate quality)
Kronstad *et al.*, 2025^(27)^	*	*	*			*			NA			4 (moderate quality)
Varre *et al.*, 2025^(49)^	*		*			*			NA			3 (low quality)
Zheng *et al.*, 2025^(50)^	*	*	*			*			NA			4 (moderate quality)

* Indicates that the systematic review article possesses this quality and is awarded one point on the AMSTAR scale; NA = ‘Not applicable’ and it is placed for the ‘Methods used to combine appropriate’ because this can only be assessed in systematic reviews that include meta-analysis and a pooled estimate.

## Discussion

This umbrella literature review synthesised evidence from thirty-three systematic reviews to explore the multifactorial influences of cultural, socio-economic, psychological stressors, environmental and physiological factors on obesity and weight gain among immigrants to high-income countries. The findings reinforce the complexity of obesity as a public health issue, particularly within migrant populations, and align with the broader literature that positions obesity as a multifaceted condition influenced by biological, behavioural and contextual determinants^(1,2)^. Importantly, the evidence shifts the interpretation of immigrant obesity away from individual lifestyle choices towards structural and contextual processes that govern exposure, opportunity and adaptation.

Specifically, this umbrella review showed that immigrants to high-income countries are at increased risk of obesity and related cardiometabolic conditions over time, despite often arriving with a health advantage – a phenomenon known as the healthy migrant effect. This advantage diminishes as immigrants acculturate to the host country environments, which are frequently characterised by obesogenic dietary patterns, sedentary lifestyles and structural barriers to health-promoting resources^(2,8,11)^. This aligns with both the reviews included in this umbrella review and the wider literature. For instance, Shah *et al.* (2023) showed that female migrants in the United Arab Emirates with 5 years of residence were twice as likely to be overweight or obese, even after adjusting for age^(53)^.

Cultural factors, particularly dietary acculturation and duration of residence, emerged as consistent predictors of weight gain. Immigrants often transition from traditional diets rich in fiber and micronutrients to Western diets high in fats, sugars and processed foods, referred to as the Nutrition Transition Theory^(54)^. This shift is not uniform but rather the influence depends on the context, generation, gender and socio-economic position. For example, our umbrella review has shown that the shift in diet follows intergenerational dynamics and is especially pronounced among second-generation immigrants and younger individuals, who are more susceptible to the influences of host country food environments and social norms^(36,47)^. Consistent with our findings, Jäger *et al.* (2022) reported that, after adjusting for socio-demographic factors, second generation – but not first-generation – immigrants in Germany had higher BMI and obesity risk than the domestic-born population^(55)^.

Furthermore, acculturation, commonly measured by length of residence or language proficiency, was generally associated with higher BMI and adiposity, though effects were non-linear and context-specific^(47,49)^. Our umbrella review also showed that whilst Western dietary adoption increased exposure to processed foods, many immigrants retained key elements of traditional diets, reflecting bicultural rather than assimilative eating patterns^(35,50)^. Cultural retention was neither uniformly protective nor harmful, as traditional practices could support healthy eating or, alternatively, promote higher energy intake and reduced physical activity^(27)^. Similar complexity was observed by Li *et al.* (2023) among Chinese immigrants in Portugal, where dietary mixing increased with length of residence without extreme Westernisation^(56)^.

Socio-economic stressors – including lower socio-economic status, job instability, food insecurity and limited access to healthcare –were also strongly associated with a higher obesity risk among immigrants. Food insecurity emerged as a key mechanism, with prevalence reaching up to 70 % in some refugee and recent migrant populations and was paradoxically associated with both undernutrition and overweight due to reliance on inexpensive energy-dense, nutrient-poor foods^(37,50)^. Psychological stressors – including acculturative stress, discrimination, job insecurity and precarious legal status – were also consistently linked to unhealthy diets, emotional eating, reduced physical activity and poor sleep, reinforcing bidirectional feedback loops between mental health and obesity, particularly among women and refugees^(11,21,35,38)^.

Socio-economic stressors operated through place-based factors such as neighbourhood deprivation and residential segregation were also evident. Evidence from the USA showed that immigrants and ethnic minorities in high-poverty or segregated areas face greater exposure to fast food, limited recreational resources and chronic stress, reinforcing obesity disparities independent of individual behaviours^(44,45)^. Consistently, Bell *et al.* (2019) linked county-level racial inequalities in poverty, unemployment and homeownership to higher obesity rates through reduced access to grocery stores and increased fast-food availability, highlighting the role of structural racism in shaping obesogenic environments^(57)^.

Environmental and physiological factors also contributed to weight gain following migration. Multiple reviews^(10,51)^ indicate that immigrants from low- and middle-income countries often undergo a rapid shift from nutritionally constrained environments to calorie-dense, sedentary contexts in high-income countries, consistent with Nutrition Transition Theory^(54)^. This transition disproportionately affects metabolically vulnerable groups, such as South Asian and African immigrants, who experience increased central adiposity and metabolic risk at relatively modest BMI gains^(31,32)^.

Physical climate acts primarily as a behavioural modifier rather than a direct cause of obesity. Cold temperatures, limited daylight and poor walkability – especially in Nordic contexts – restrict physical activity and access to healthy foods, disproportionately affecting women and older adults^(27,35,36)^. These constraints interact with transport, occupational patterns and neighbourhood design to reinforce sedentary lifestyles. Reviews also reported micronutrient deficiencies, particularly vitamin D and folate, among pregnant women and refugees due to reduced sun exposure and dietary change^(33,34)^. The cumulative metabolic effects of these exposures help explain higher obesity risk with longer duration of residence across many populations^(28,29,47)^. Similar to our findings, Katare & Chakrovorty (2019) found an association between the surrounding local environmental factors and the BMI of immigrants in the USA^(58)^.

Despite the breadth of the reviewed evidence, several critical gaps remain. First, many of the studies reviewed by the thirty-three included systematic reviews were cross-sectional, limiting the ability to infer causality or track changes over time. Longitudinal studies are needed to better understand the temporal dynamics of weight gain and the cumulative effects of acculturation, socio-economic disadvantage and environmental exposure. Studying the temporality aspect in more depth is important given that the length of stay in the host country was a significant contributor towards weight gain and obesity among immigrants to high-income countries.

Second, there is a lack of standardised measures for key constructs such as acculturation, dietary change, psychological stress, food insecurity and physical activity. This heterogeneity in measurement limits comparability across studies and may obscure important subgroup differences. For example, while some studies found that acculturation was associated with increased BMI^(10,47)^, others reported protective effects among women^(27,29)^, suggesting that gender, cultural norms and body image perceptions may moderate this relationship. Similarly, refugees did not uniformly exhibit higher obesity prevalence than host populations in meta-analyses, yet subgroup analyses revealed divergent risks by sex and region^(30)^. Heterogeneity was also found with respect to pre-migration exposures, selection processes, legal status, labour market integration and welfare regimes across host countries. For example, Nordic welfare states, while offering universal healthcare, were not immune to obesity inequalities, particularly among marginalised immigrant women facing cultural, linguistic and climatic barriers^(27)^. These contextual contrasts reinforce the importance of analysing immigrant obesity through a comparative, systems level lens, rather than attributing outcomes to migration status alone.

Third, the role of climate and physical environment remains underexplored. While some reviews touched on vitamin D and folate deficiency and cold-weather barriers to physical activity and access to healthy food^(27,33–36)^, few studies systematically examined how climatic transitions affect metabolic health, dietary behaviours or psychological well-being. This is a particularly significant gap that requires further exploration beyond accessibility and vitamins deficiency dimensions given the growing number of migrants relocating from tropical or subtropical regions to temperate or colder climates. Additionally, bridging the connection between fluctuations in environmental conditions such as climatic/weather factors and air pollution between immigrants’ sending and receiving countries and how it impacts weight gain and obesity across the immigrant population would form an interesting future project.

Fourth, there is limited attention to intersectionality and heterogeneity within immigrant populations. Factors such as ethnicity, religion, migration status (e.g. refugee *v*. economic migrant), age at arrival and sex were often mentioned but rarely analysed in depth. For example, immigrant populations were often treated as homogenous groups, with insufficient disaggregation by ethnicity, sex or migration history. This lack of granularity may mask important variations in obesity risk and resilience across different subgroups of immigrants, hence, requiring further research.

Finally, methodological quality varied across the included systematic reviews. While three meta-analyses were rated as high quality and three as moderate quality, the majority of the systematic reviews did not conduct meta-analysis to pool estimates and lacked transparency in study selection, quality appraisal and conflict of interest reporting. This highlights the need for more rigorous and transparent synthesis methods in future research.

### Implications for future research

More research that adopts different study designs such as longitudinal and mixed-methods designs is needed to capture the dynamic and context-dependent nature of weight gain and obesity among immigrants to high-income countries. Standardised tools for measuring acculturation, dietary change, physical activity, psychosocial stress and food security and accessibility are to be developed and validated across diverse populations of immigrants and host countries. Additionally, studies should incorporate intersectional frameworks to examine how overlapping ethnic identities and migration histories as well as structural inequalities shape health outcomes such as weight gain and obesity.

Furthermore, there is a need for more research on the physiological and behavioural/psychological impacts of climate change (e.g. differences in climatic/weather factors and air pollution between immigrant sending/receiving countries) and geographic relocation. For example, investigating how temperature, sunlight exposure and seasonal variation for immigrants from the origin to the destination country influence metabolism, physical activity and mental health could yield valuable insights into the environmental determinants of weight gain and obesity.

Finally, future studies should explore protective cultural practices and resilience factors that may buffer against weight gain and obesity. This could be done using mixed-methods study designs involving both quantitative and qualitative analysis. Understanding how some immigrant groups maintain healthy behaviours despite adverse conditions could help inform culturally tailored interventions and public health promotion initiatives.

### Policy and practice implications

This umbrella literature review provided an overview on the impacts of cultural, socio-economic and climate variations on obesity and weight gain amongst immigrants to high-income countries, which would help in guiding public health policy interventions and practice. First, early intervention is critical. Given that significant weight gain often occurs within the first decade of residence, host countries should implement culturally sensitive orientation programmes that promote healthy eating, physical activity and mental well-being from the outset of the migration experience. This could include providing culturally appropriate nutrition education, improving access to healthy foods and designing interventions that consider language, literacy and cultural norms whether at schools, workplaces or public spaces and community events.

Second, policies should address structural barriers to health, including food insecurity, housing instability, employment instability, transportation/accessibility infrastructure and limited access to culturally appropriate healthcare. Expanding access to affordable, nutritious and culturally familiar foods –particularly in immigrant-dense neighbourhoods – could help mitigate dietary acculturation and its negative health consequences.

Third, mental health services should be integrated into obesity prevention strategies, particularly for high-risk groups such as refugees, women and low-income/low-education immigrants. Addressing acculturation stress, discrimination and social isolation through urban planning and community-based support programmes could reduce the psychological risk factors for weight gain and obesity and promote a more active lifestyle among immigrant populations. For example, community-based programmes that provide cultural local food stores, safe walking paths, gardens/parks initiatives and recreational facilities could help in this regard.

Finally, public health surveillance systems should disaggregate data by migration status, ethnicity and other relevant variables to better monitor trends and target interventions. This would support more equitable and effective health policies that recognise the unique challenges faced by specific immigrant sub-groups.

### Strengths and limitations of the conducted umbrella literature review

This umbrella review synthesises comprehensively findings from thirty-three systematic and scoping reviews published over the past decade, examining obesity and weight gain among immigrants to high-income countries. Using a thematic framework and methodological quality assessment, it provides a multilevel perspective and evaluates transparency and confidence in the evidence.

However, this umbrella literature review exhibits some limitations including: (1) restriction to English-language publications, potentially excluding relevant studies from non-English contexts; (2) reliance of umbrella reviews by design on the scope and quality of existing reviews, which may overrepresent certain populations (e.g. US-based) and underrepresent others; (3) exclusion of intervention-focused reviews, limiting causal inference and 4) dependence on self-reported BMI, obesity and acculturation measures in most of the reviewed studies, which may introduce heterogeneity and reduce methodological quality.

### Conclusion

This umbrella review synthesised evidence from thirty-three systematic reviews to examine cultural, socio-economic and environmental drivers of obesity and weight gain among immigrants to high-income countries. Although immigrants often arrive healthier than native-born populations, this advantage declines over time due to dietary acculturation, longer residence, socio-economic stressors, food insecurity and limited access to culturally appropriate services. Environmental factors, including cold climates and reduced daylight, may indirectly limit physical activity and healthy food access. However, the literature is constrained by methodological inconsistencies and limited longitudinal, mixed-methods and intersectional analyses. Targeted, culturally sensitive public health strategies are therefore essential to address the structural determinants of weight gain/obesity and promote health equity among immigrants to high-income countries.

## Supporting information

10.1017/S1368980026102687.sm001 Abed Al Ahad and Fenyk supplementary material Abed Al Ahad and Fenyk supplementary material

## Supplementary material

For supplementary material accompanying this paper visit https://doi.org/10.1017/S1368980026102687

## Table 1. Long description

The table presents a detailed summary of thirty-three systematic review articles included in the synthesis of an umbrella literature review. It provides key findings and thematic classifications of these articles, offering a comprehensive overview of their content and contributions. The table is divided into two sections: Table 1 summarizes the key findings and thematic classification, while Table 2 offers a more detailed systematic summary of the characteristics of the thirty-three review articles.

Navigate back to Table 1..

## Table 2. Long description

The table presents a systematic summary of 33 review articles included in an umbrella literature review. It categorizes the key findings and thematic classifications of these articles. The table is organized into multiple columns and rows, detailing the characteristics and summaries of each review article. The columns likely include headers such as the title of the review article, key findings, thematic classification, and other relevant details. Each row corresponds to a different review article, providing a concise overview of its content and contributions to the umbrella review.

Navigate back to Table 2..

## Figure 1 Long description

A pie chart illustrates the distribution of three themes across 33 review articles. The chart is divided into four segments. The largest segment, colored in blue, represents 55 percent and is labeled ‘Socioeconomic stressors and psychological mediators.’ The second-largest segment, colored in green, represents 39 percent and is labeled ‘Socioeconomic stressors, psychological mediators, and cultural factors.’ Two smaller segments, each representing 3 percent, are colored in dark blue and orange. The dark blue segment is labeled ‘Socioeconomic stressors and psychological mediators,’ and the orange segment is labeled ‘Cultural factors.’ The chart highlights the dominant presence of socioeconomic stressors and psychological mediators in the reviewed articles.

Navigate back to Figure 1.

## Table 3. Long description

The table presents a summary quality assessment of six systematic reviews that included meta-analysis, evaluated using the A Measurement Tool to Assess Systematic Reviews (AMSTAR) tool. The table has 11 rows and 6 columns, with each column representing a different systematic review and each row representing a specific AMSTAR question. The columns are labeled with the names of the reviews and their publication years: Amiri, 2021; Mensah et al., 2022; Lopez et al., 2023; Paixão et al., 2023; Kibibi et al., 2024; and Mendez 2025. The rows are labeled with AMSTAR questions such as ‘A priori design provided,’ ‘Duplicate study selection and data extraction,’ ‘Comprehensive literature search,’ and others. Each cell indicates whether the corresponding review met the criteria for the respective AMSTAR question, marked with an asterisk if met. The total scores for each review are provided at the bottom, with three reviews achieving high quality (scores of 8 or 9) and three scoring moderate quality (scores of 6 or 7). The table highlights the rigorous methodology of these reviews, including comprehensive literature searches, duplicate data extraction, and appropriate use of quality assessments and statistical methods. However, it also notes weaknesses such as limited reporting of publication bias and conflict of interest.

Navigate back to Table 3..

## Table 4. Long description

The table presents a quality assessment of 27 systematic review articles that did not include a meta-analysis, using the AMSTAR criteria. It includes columns for various AMSTAR questions such as ‘A priori design provided’, ‘Duplicate study selection and data extraction’, ‘Comprehensive literature search’, ‘Publication status as an inclusion criteria’, ‘List of studies included and excluded provided’, ‘Characteristics of included studies provided’, ‘Quality assessment’, ‘Quality used appropriately’, ‘Methods used to combine appropriate’, ‘Publication bias assessed’, and ‘Conflict of interest stated’. Each row corresponds to a different systematic review article, with checkmarks indicating whether the criteria were met. The total score for each article is provided, ranging from low quality (score 3) to moderate quality (scores 4-7), with only one article rated as high quality (score 8). Notable trends include common strengths such as clear inclusion criteria and comprehensive searches, and frequent limitations such as lack of duplicate data extraction and incomplete lists of included or excluded studies.

Navigate back to Table 4..
