# Supplementary material for: Obesity and the experienced cultural, economic and climate variations among immigrants to high-income countries: An umbrella review of the literature
Source: Public Health Nutr. 2026 May 28;29(1):e123. doi: 10.1017/S1368980026102687 (PMC13434502; doi:10.1017/S1368980026102687)
Supplement: Supplementary file 1 [file S1368980026102687sup001.docx]

**Supplementary Table 1.** The search algorithms used in PubMed to capture literature that addressed the intersection of migration, obesity, socioeconomic and environmental determinants.

| Search algorithms | Number of systematic reviews identified in Pubmed | Number of systematic reviews identified in Web of Science | Number of systematic reviews identified in Scopus |
| --- | --- | --- | --- |
| Immigra* AND Weight Gain AND Review [PT] | 7 | 11 | 11 |
| migrat* AND Weight Gain AND Review [PT] | 12 | 11 | 23 |
| refuge* AND Weight Gain AND Review [PT] | 2 | 1 | 5 |
| Obesity AND Immigra* AND Review [PT] | 55 | 98 | 114 |
| Obesity AND migrat* AND Review [PT] | 301 | 484 | 227 |
| Obesity AND refuge* AND Review [PT] | 22 | 27 | 36 |
| Climate AND Obesity AND immigra* AND Review [PT] | 0 | 1 | 1 |
| Climate AND Obesity AND migrat* AND Review [PT] | 7 | 7 | 5 |
| Culture AND Weight Gain AND Immigra* AND Review [PT] | 5 | 2 | 2 |
| Culture AND Weight Gain AND migrat* AND Review [PT] | 2 | 0 | 1 |
| Migrating Populations AND Weight Gain AND Review [PT] | 6 | 3 | 1 |
| Environment AND Obesity AND Immigra* AND Review [PT] | 12 | 14 | 20 |
| Environment AND Obesity AND migrat* AND Review [PT] | 49 | 38 | 16 |
| Environment AND Obesity AND refuge* AND Review [PT] | 4 | 3 | 3 |
| "cultural factors" OR acculturation OR "dietary changes"[TIAB] OR "lifestyle changes"[TIAB] OR "social environment"[TIAB] OR "food preferences"[TIAB] OR tradition[TIAB] AND Obesity AND immigra* | 15 | 35 | 36 |
| "cultural factors" OR acculturation OR "dietary changes"[TIAB] OR "lifestyle changes"[TIAB] OR "social environment"[TIAB] OR "food preferences"[TIAB] OR tradition[TIAB] AND Obesity AND migrat* | 11 | 19 | 24 |
| "cultural factors" OR acculturation OR "dietary changes"[TIAB] OR "lifestyle changes"[TIAB] OR "social environment"[TIAB] OR "food preferences"[TIAB] OR tradition[TIAB] AND Obesity AND refuge* | 5 | 7 | 9 |
| economic factors OR socioeconomic OR poverty OR income OR employment OR food security[TIAB] OR affordability AND Obesity AND Immigra* AND Review [PT] | 31 | 63 | 59 |
| economic factors OR socioeconomic OR poverty OR income OR employment OR food security[TIAB] OR affordability AND Obesity AND migrat* AND Review [PT] | 30 | 60 | 46 |
| economic factors OR socioeconomic OR poverty OR income OR employment OR food security[TIAB] OR affordability AND Obesity AND refuge* AND Review [PT] | 13 | 17 | 15 |
| physical environment OR "built environment"[TIAB] OR "access to healthy food"[TIAB] OR "physical activity"[TIAB] OR "sedentary behavior"[TIAB] OR "green spaces"[TIAB] OR "walkability"[TIAB] AND Obesity AND Immigra* [TIAB] AND Review [PT] | 7 | 29 | 31 |
| physical environment OR "built environment"[TIAB] OR "access to healthy food"[TIAB] OR "physical activity"[TIAB] OR "sedentary behavior"[TIAB] OR "green spaces"[TIAB] OR "walkability"[TIAB] AND Obesity AND migrat* [TIAB] AND Review [PT] | 14 | 25 | 21 |
| physical environment OR "built environment"[TIAB] OR "access to healthy food"[TIAB] OR "physical activity"[TIAB] OR "sedentary behavior"[TIAB] OR "green spaces"[TIAB] OR "walkability"[TIAB] AND Obesity AND refuge* [TIAB] AND Review [PT] | 1 | 7 | 7 |
| Air Pollution AND Obesity AND Immigra* AND Review [PT] | 1 | 2 | 2 |
| Air Pollution AND Obesity AND migrat* AND Review [PT] | 5 | 7 | 7 |
| Air Pollution AND Obesity AND refuge* AND Review [PT] | 1 | 1 | 1 |
| Air quality AND Obesity AND Immigra* AND Review [PT] | 1 | 0 | 1 |
| Air quality AND Obesity AND migrat* AND Review [PT] | 5 | 1 | 0 |
| Air quality AND Obesity AND refuge* AND Review [PT] | 1 | 0 | 0 |
| Temperature* AND Obesity AND migrat* AND Review [PT] | 2 | 4 | 2 |
| Heat* AND Obesity AND Immigra* AND Review [PT] | 1 | 0 | 1 |
| Heat* AND Obesity AND migrat* AND Review [PT] | 4 | 2 | 3 |
| Heat* AND Obesity AND refuge* AND Review [PT] | 0 | 1 | 1 |
| Cold* AND Obesity AND migrat* AND Review [PT] | 1 | 1 | 2 |
| Duplicates between the search codes per each search database that are removed | 270 | 400 | 384 |
| Total number of identified studies | 363 | 581 | 349 |
| Duplicates between the search databases that are removed | 384 | | |
| Total number of identified studies that underwent title and abstract screening | 909 | | |
| Total number of studies that underwent full text screening | 82 | | |
| Total number of articles that are included in the narrative synthesis | 33 | | |

Note that we tried all combinations of immigra*, migrat*, and refuge*, but when a certain search reveals zero results, it is not reported in the table. The reasons for excluding articles after full-text screening were as follows: 1) the review is conducted in a non-systematic narrative way (with no clear methodology section and systematic search algorithm); 2) the review summarised only qualitative research; 3) the review focused solely on interventions regrading obesity rather than determinants; 4) the review focused only on youth and children population rather than adults; 5) the review focused on other non-communicable diseases such as cardiovascular diseases and diabetes with no direct focus on obesity and weight gain; 6) the review did not focus on immigrants from middle and low-income countries to high income countries, but rather assessed obesity/weight gain globally or in the general population rather than immigrant population; 7) the review was a conference proceeding or a commentary rather than a full systematic review article; and one article focused on biology microbiome changes post immigration rather than obesity/weight change per say (Shad, N. S., Shaikh, N. I., & Cunningham, S. A. (2024). Migration spurs changes in the human microbiome: a review. *Journal of Racial and Ethnic Health Disparities*, *11*(6), 3618-3632).
